# Investigating the Effects of Long-Term Fine Particulate Matter Exposure on Autism Spectrum Disorder Severity: Evidence from Multiple Analytical Approaches

**DOI:** 10.3390/toxics13110922

**Published:** 2025-10-28

**Authors:** Jianrui Dou, Kaiyue Zhang, Ruijin Xie, Hua Xu, Qiyang Pan, Xue Xiao, Yufan Luo, Shengjie Xu, Wei Xiao, Dongqin Wu, Bing Wang, Linpei Zhang, Chenyu Sun, Yueying Liu

**Affiliations:** 1 Department of Occupational Safety and Health, Center for Disease Control and Prevention of Yangzhou, Yangzhou 225000, China; 2 School of Medicine, Jiangnan University, Wuxi 214122, China; 3 Department of Psychiatry and Psychology, Mayo Clinic, Rochester, MN 55905, USA; 4 Key Laboratory of Carbohydrate Chemistry and Biotechnology, Ministry of Education, School of Biotechnology, Jiangnan University, Wuxi 214122, China; 5 Division of Public Health, Infectious Diseases, and Occupational Medicine, Mayo Clinic, Rochester, MN 55905, USA; 6 Mayo Clinic School of Graduate Medical Education, Mayo Clinic College of Medicine and Science, Rochester, MN 55905, USA; 7 School of Public Health, University of Minnesota-Twin Cities, Minneapolis, MN 55455, USA

**Keywords:** neurotoxicity, co-exposure, PM_2.5_, network toxicology, autism

## Abstract

With rapid industrial expansion, air pollution’s adverse neurological effects have gained increasing attention. Children face a greater risk of neurological damage because of their higher breathing rates, developing brains, and limited ability to detoxify harmful substances. Fine particulate matter has been identified as a primary neurotoxic contributor affecting developing brains. Strong evidence connects environmental pollutant exposure to the prevalence of Autism Spectrum Disorder (ASD), a neurodevelopmental condition marked by lasting difficulties with social communication and interaction. This study explores the association between long-term PM_2.5_ exposure and ASD symptom exacerbation, investigating underlying mechanisms. We hypothesize that long-term PM_2.5_ exposure exacerbates ASD symptoms through neuroinflammatory activation, leading to neuronal damage and impaired synaptic plasticity. Our investigation employs three complementary approaches: First, integrated analysis combining Global Burden of Disease data with Mendelian randomization demonstrates a significant association between PM_2.5_ exposure and increased ASD severity risk. Second, utilizing the China High-Resolution Air Pollution Database in conjunction with cohort studies, we provide evidence that ambient air pollution substantially influences autism severity, with PM_2.5_ identified as the predominant environmental determinant. Third, through network toxicology, single-cell transcriptomics, and animal experimentation, we demonstrate that chronic PM_2.5_ exposure exacerbates valproic acid-induced autism-like behaviors in murine models, identifying CTNNB1, PTEN, CCR2, AKT1, and mTOR as potential core mediating genes. Importantly, these findings represent preliminary results. Several potential confounding factors such as co-exposure to other pollutants and socioeconomic variables have not been fully addressed. While our multi-modal approach provides converging lines of evidence, further validation in larger, more diverse populations with refined control of confounders will be essential to establish causality and elucidate mechanisms. Nonetheless, these early insights advance our understanding of PM_2.5_-induced neurotoxicity in the context of ASD and offer timely, albeit preliminary, evidence to inform public health policy.

## 1. Introduction

As industrial production expands, air pollution has become a major concern. A growing body of scientific evidence now links common pollutants to significant adverse health outcomes, particularly those affecting the neurological system [1,2]. Among the general population, children are especially susceptible to the neurological impact of air pollution due to their higher respiratory rates, ongoing brain development, and less efficient detoxification mechanisms [3]. Furthermore, the blood–brain barrier, which serves as a protective shield in adults, is more permeable during early life, thereby allowing harmful particles and chemicals to penetrate sensitive neural tissues more easily [4].

Air pollution is a complex and variable mixture of gases and particles. Among its components, fine particulate matter with a diameter of 2.5 μm or less (PM_2.5_) has been identified by scientists and public health officials as a primary contributor to neurological impact on the developing brain [5]. Among various pediatric neurological disorders, mounting evidence indicates a robust association between environmental pollution and Autism Spectrum Disorder (ASD), likely because pollutants can trigger the neuroinflammation that is a key characteristic of the condition [6].

The diagnosis of ASD is based on two core features: lasting difficulties with social skills and communication, as well as the presence of restricted, repetitive patterns of behavior, interests, or activities [7]. At the molecular and genetic level, ASD is recognized as a highly heterogeneous neurodevelopmental disorder with strong heritability. Large-scale genomic studies have identified hundreds of risk genes, many of which converge on shared biological pathways critical for synaptic function, neuronal connectivity, and cortical development [8]. Notably, recurrent disruptions in genes such as SHANK3, NLGN3/4, NRXN1, PTEN, and CTNNB1 have been consistently linked to ASD pathogenesis [9]. These genes regulate key signaling cascades, including the PI3K/AKT/mTOR and Wnt/β-catenin pathways, that govern neuronal growth, dendritic arborization, and activity-dependent synaptic plasticity [10,11]. Moreover, epigenetic dysregulation, including altered DNA methylation and histone modification patterns in response to environmental stimuli, has emerged as a critical interface between genetic susceptibility and external risk factors in ASD [10,11]. This gene–environment interplay underscores the plausibility that environmental toxicants, such as PM_2.5_, may exacerbate ASD phenotypes by perturbing these same vulnerable neurodevelopmental pathways. Clinically, individuals with ASD commonly exhibit cognitive deficits in social cognition, attention, and working memory, as well as anxiety-related behaviors, which contribute substantially to impairments in adaptive functioning and quality of life [12,13]. Epidemiological studies have consistently demonstrated that prenatal exposure to elevated concentrations of ambient air pollutants is associated with increased risk of ASD in offspring, with mechanistic plausibility supported by neuroinflammatory cascades and epigenetic modifications [14]. However, investigations regarding whether sustained environmental PM_2.5_ exposure may exacerbate symptoms in children with ASD, as well as its potential mechanisms, remain limited. Therefore, the primary aim of this study is twofold: to explore the link between sustained PM_2.5_ exposure and the exacerbation of ASD symptoms, and to investigate the underlying mechanisms.

In this study, we hypothesize that sustained environmental PM_2.5_ exposure may exacerbate ASD symptoms in children through interconnected pathophysiological mechanisms involving activation of neuroinflammatory responses, subsequently leading to neuronal damage and impaired synaptic plasticity, particularly long-term potentiation. Subsequently leading to neuronal damage and impaired long-term potentiation. Our investigation provides novel insights across three complementary research domains: First, through integrated analysis combining Global Burden of Disease (GBD) data on pediatric ASD and Mendelian randomization approaches, we demonstrate that PM_2.5_ exposure may associated with increased ASD severity risk. Second, leveraging the China High-resolution Air Pollution (CHAP) Database in conjunction with the pilot cohort study of children with ASD and employing three distinct co-exposure statistical methodologies, we provide evidence that ambient air pollution significantly affects autism severity, with PM_2.5_ emerging as the primary environmental determinant influencing ASD symptom manifestation. Third, through an integrative approach combining human network toxicology, single-cell transcriptomic analyses, and animal experiments, we demonstrate that chronic environmental PM_2.5_ exposure significantly exacerbates valproic acid (VPA)-induced autism-like behaviors in murine models. Furthermore, our molecular analyses identify CTNNB1, PTEN, CCR2, AKT1, and mTOR as core target genes mediating the neurological impact of long-term PM_2.5_ exposure in the context of ASD pathophysiology. Collectively, our findings advance the mechanistic understanding of PM_2.5_-induced neurotoxicity in ASD and provide critical evidence to inform evidence-based public health policies and clinical intervention strategies targeting air pollution-related neurological disorders. This comprehensive investigation not only elucidates the molecular underpinnings of PM_2.5_-mediated neurotoxicity but also identifies promising therapeutic targets for future intervention development.

## 2. Materials and Methods

### 2.1. Global Burden of Disease Analysis on Pediatric Autism Spectrum Disorder

We first conducted a Global Burden of Disease analysis on pediatric autism spectrum disorder based on our previous studies [15,16]. Briefly, we assessed and compared the disease burden in children aged 0–14 years across countries and territories in 2021. Age-standardized DALY rates were computed via the direct standardization method, following established protocols from prior research. The age-specific rates for the <5, 5–9, and 10–14 age groups were weighted using a truncated WHO 2000–2025 Standard Population, which was normalized to sum to 100% within this specific age range (Supplemental Table S1) [17]. Additionally, correlation analysis was employed to examine the relationship between age-standardized DALY rates of ASD (among children aged 0–14 years) and annual average PM_2.5_ concentrations across countries and territories. For this study, yearly average PM_2.5_ concentrations (μg/m^3^) for all countries and territories in 2021 were sourced from the 2021 World Air Quality Report published by IQAir (https://www.iqair.com/, accessed on 25 September 2025).

### 2.2. Pilot Cohort Description and Study Design

In this study, we implemented an observational cohort design to further examine the potential impact of complex air pollution mixtures on ASD symptomatology, building upon findings from our prior research [15,16,18,19]. Briefly, we established an Pilot observational cohort comprising 321 children diagnosed with ASD who were newly admitted to the Department of Paediatrics at the Affiliated Hospital of Jiangnan University between January 2021 and December 2024 (see Supplemental Figure S1). To minimize potential confounding from geographic variability in air pollution exposure, all participating children were required to have resided in the same residential community for at least three consecutive years prior to enrollment and to have been born in Wuxi. This observational cohort study received ethical approval from the Ethics Committee of the Affiliated Hospital of Jiangnan University (Approval No. LS2021JN08024R1). The severity of ASD symptoms was assessed using the Childhood Autism Rating Scale (CARS), with scores systematically recorded for each participant. CARS is a 15-item scale in which a trained professional rates a child’s behavior on a scale from 1 (within normal limits) to 4 (severely abnormal), used to determine whether a child falls on the autism spectrum and to assess the severity of their symptoms (Supplemental Information S1). The basic demographic and clinical characteristics of these 321 children diagnosed with ASD are presented in Supplemental Table S2.

### 2.3. Community-Level Participants Air Pollution Mixture Levels Calculation

To assess community-level air pollution mixture exposure among participants, we first geocoded the residential addresses to obtain longitude and latitude coordinates using the Baidu Map Service (https://lbs.baidu.com/maptool/getpoint, accessed on 25 September 2025). Subsequently, based on Euclidean distance approximation in latitude-longitude space, we extracted concentration data for key pollutants, including PM_10_, PM_2.5_, CO, SO_2_, and NO_2_ from the China High-resolution Air Pollution Database (https://zenodo.org/communities/chap/, accessed on 25 September 2025) The China High-resolution Air Pollution Database (CHAP) Database has been recognized as a robust and reliable source of air pollution data for China and has been extensively utilized in numerous related studies [20]. These extracted pollutant concentrations were used to characterize the community-level air pollution mixture exposure for all participants [21].

### 2.4. Multiple Co-Exposure Statistical Models Approaches

Three statistical models were employed to analyze the health effects of multiple simultaneous exposures, with the aim of identifying the most impactful chemicals within the mixture on health outcomes and elucidating their combined effects, as established in previous studies [22]. In all models, the exposure variables consisted of community-level annual average concentrations of five criteria air pollutants: PM_10_, PM_2.5_, CO, SO_2_, and NO_2_. The primary outcome was the total score from the Childhood Autism Rating Scale (CARS), treated as a continuous variable to reflect the dimensional nature of ASD symptom severity. To account for potential confounding, we adjusted for a priori selected covariates including age (years), sex (male/female), body mass index (BMI, kg/m^2^), and race/ethnicity. These covariates were consistently included across all three modeling frameworks to ensure comparability of results. Detailed descriptions of the three statistical models are provided in Supplemental Information S2.

### 2.5. Environmental PM_2.5_ Sample Collection

To better reflect the neurological impact of environmental PM_2.5_, we collected real-world environmental PM_2.5_ samples [23]. First, filter membranes were preconditioned in a temperature and humidity-controlled chamber for at least 24 h, then weighed on a high precision microbalance to obtain baseline mass. These filters were then mounted in PM_2.5_ samplers at selected monitoring stations, where ambient air was collected at a constant flow rate over a 14-day sampling period to selectively capture particles with diameter of ≤2.5 μm. After sampling, filters were returned to the controlled environment for 24 h of equilibration before final weighing to determine total particulate mass. Filters were then sectioned using ceramic scissors to avoid contamination and transferred into digestion vessels. A mixture of nitric and hydrochloric acids was added, followed by microwave assisted digestion to fully solubilize both particulate matter and filter matrix. The resulting digestate was lyophilized using an ultra-low-temperature freeze dryer, allowing solvent sublimation under reduced pressure. The dried residue was reconstituted and used as the environmental PM_2.5_ exposure agent in subsequent animal experiments.

### 2.6. Experimental Environmental PM_2.5_ Level Calculation

To better reflect the neurological impact of environmental PM_2.5_, we chose environmental PM_2.5_ levels based on previous studies [24,25]. The calculation of environmental PM_2.5_ levels can be summarized in the following steps briefly:

1. Daily Respiratory Frequency: A mouse’s respiratory rate is approximately 120 breaths per minute. Therefore, the total number of breaths per day is: 120 breaths/min × 60 min/hour × 24 h/day = 172,800 breaths/day.
2. Daily Respiratory Volume: The tidal volume of a single mouse breath is 0.15 mL. Therefore, the total daily respiratory volume is: 172,800 breaths/day × 0.15 mL/breath = 25,920 mL/day = 25.92 L/day
3. Conversion of Daily Respiratory Volume to Cubic Meters: 25.92 L/day ÷ 1000 L/m^3^ = 0.02592 m^3^/day.
4. Daily Inhaled Mass of PM_2.5_: The mass of PM_2.5_ inhaled per day by a mouse is: 50 μg/m^3^ × 0.02592 m^3^/day ≈ 1.3 μg/day.
5. Calculation of Dosage for Administration: Assuming a standard body weight of 20 g (0.02 kg) and intratracheal instillation every three days to reduce animal distress, the dosage is calculated as: (1.3 μg/day ÷ 0.02 kg) × 3 days ≈ 200 μg/kg per 3-day period.
6. Final Dose Adjustment: According to principles of environmental toxicology and previous studies [18,25], a 10-fold safety factor is applied to account for intra-species individual variability and potential cumulative effects. The final administered dose is therefore: (200 μg/kg × 10) ÷ 1000 μg/mg = 2 mg/kg, administered every 3 days as the low environmental PM_2.5_ exposure level.

Additionally, a dose of 5 mg/kg every 3 days was considered as the high environmental PM_2.5_ exposure level based on our previous study. Ref. [18], representing approximately 2.5-fold higher exposure to simulate urban areas with severe air pollution conditions. This intermittent, physiologically grounded dosing strategy balances environmental relevance with animal welfare considerations and experimental tractability [18].

### 2.7. Determination of Sample Size

The sample size for animal experiments was rigorously determined through formal a priori statistical power analysis to ensure sufficient statistical power for detecting meaningful biological effects while adhering to the ethical principles of the 3Rs (Replacement, Reduction, and Refinement) in animal research. Power analysis was performed using the standard formula for comparing means between two independent groups:

$$\mathrm{Sample}\;\mathrm{Size}\;=\frac{2\times {\left(Z_{\alpha /2}+Z_{\beta }\right)}^{2}\times \sigma^{2}}{d^{2}}$$

The parameters were set according to established conventions in biomedical research: A significance level (α) of 0.05, corresponding to a Z-score (Z α/2) of 1.96. A statistical power (1 − β) of 80%, corresponding to a Z-score (Z β) of 0.84. An anticipated effect size (*d*) of 1.7σ, where σ is the standard deviation. The calculation yielded n ≈ 5.43 animals per group. Following conservative practice and accounting for potential attrition, this value was rounded up to n = 6 animals per group. This sample size balances the ethical imperative of the Reduction principle with the statistical requirement for adequate power to detect biologically relevant effects. The Reduction principle advocates using the minimum number of animals necessary to achieve valid scientific objectives. This approach minimizes animal use while maintaining methodological rigor and reproducibility. Furthermore, this sample size is consistent with established practices in neuroscience research, where n = 6 per group has been validated as appropriate for detecting significant neurobiological effects in rodent models.

### 2.8. Animal Experimental Design

To determine how sustained exposure to environmental PM_2.5_ affects the brain in relation to ASD symptoms, we performed controlled animal studies that specifically assessed cognitive function and anxiety-like behaviors using established protocols [15,16,18,26]. All animal experiments were performed in strict compliance with the protocol approved by the Animal Ethics Committee of Jiangnan University (Approval No. JN.No20240930c0230120[517]). Male C57BL/6J mice were maintained under standard laboratory housing conditions and subsequently bred for the study. Pregnancy was verified by the presence of vaginal plugs, with the day of plug detection designated as embryonic day 0 (E0). On embryonic day 12.5 (E12.5), pregnant dams received a single intraperitoneal injection of valproic acid (VPA; 500 mg/kg body weight, Sigma-Aldrich, St. Louis, MO, USA) dissolved in 0.9% sterile saline to induce ASD-like phenotypes in offspring. Control dams received an equivalent volume of sterile saline vehicle alone. At 4 weeks of age (postnatal day 28), male offspring exhibiting ASD-like behaviors following prenatal VPA exposure were selected and randomly allocated into experimental groups (n = 6 per group) [26,27]: 1. Control group (n = 6): Offspring from saline-treated dams receiving intratracheal instillation of sterile saline every 3 days for 90 days. 2. ASD group (n = 6): Offspring from VPA-treated dams receiving intratracheal instillation of sterile saline every 3 days for 90 days. 3. ASD + Low PM_2.5_ group (n = 6): Offspring from VPA-treated dams receiving intratracheal instillation of 2 mg/kg environmental PM_2.5_ every 3 days for 90 days to simulate chronic low-level environmental exposure. 4. ASD + High PM_2.5_ group (n = 6): Offspring from VPA-treated dams receiving intratracheal instillation of 5 mg/kg environmental PM_2.5_ every 3 days for 90 days to simulate chronic high-level environmental PM_2.5_ exposure. The 3-day interval between exposures was designed to permit partial recovery while maintaining cumulative inflammatory burden, a commonly employed strategy to avoid acute toxicity while inducing chronic effects. The 90-day exposure duration was selected based on our previous studies to mimic long-term environmental exposure [26]. Both Control and ASD groups underwent identical sham intratracheal instillations with sterile saline, matched in volume, frequency, and handling to PM_2.5_-exposed groups, to control for potential confounding effects of the instillation procedure.

### 2.9. Statistical Analysis

Data are presented as mean ± standard deviation (SD). We assessed data normality using the Shapiro–Wilk test. Depending on the distribution, we compared groups using either parametric tests (unpaired *t*-test, one/two-way ANOVA with Tukey’s post hoc test) or non-parametric tests (Mann–Whitney U, Kruskal–Wallis). All analyses were performed in GraphPad Prism 9.3, with statistical significance defined as *p* < 0.05. To facilitate transparency and replication of our findings, the complete code for our analyses and visualizations are available through website (https://github.com/PediatricLab-Jiangnan/Autism-Multiapproch, accessed on 20 September 2025), with a detailed methodology available in the Supplementary Methods Section.

## 3. Results

### 3.1. Air Pollution May Be Associated with Increased ASD Severity Risk

**Figure 1:**
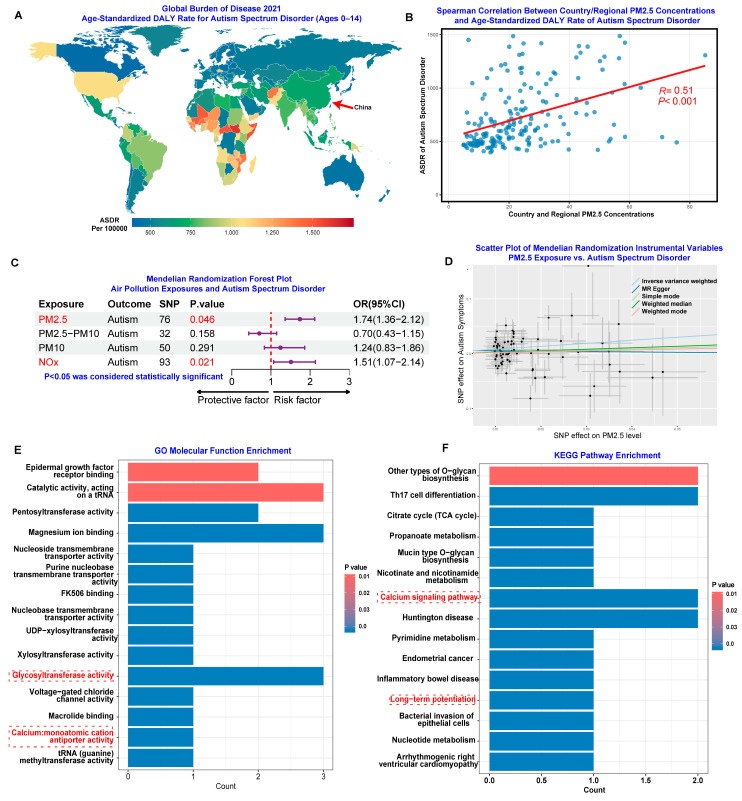
Association Between Global Autism Spectrum Disorder (ASD) Burden and Air Pollution. (**A**) World map showing the age-standardized Disability-Adjusted Life Year (DALY) rate of Autism Spectrum Disorder per 100,000 population (ages 0–14), based on Global Burden of Disease 2021 data. (**B**) Spearman correlation analysis between country/regional PM_2.5_ concentrations and age-standardized DALY rates for ASD. (**C**) Mendelian Randomization of the forest plot evaluating the potential relationship between air pollution exposures (PM_2.5_, PM_2.5_–PM_10_, PM_10_, and NO_x_) and ASD risk. (**D**) Scatter plot of Mendelian Randomization instrumental variables, illustrating the potential positive effect of PM_2.5_ exposure on ASD symptoms. (**E**) Gene Ontology (GO) molecular function enrichment analysis based on single nucleotide polymorphisms (SNPs) used as instrumental variables in the Mendelian Randomization analysis using SNPnexus database. (**F**) Kyoto Encyclopedia of Genes and Genomes (KEGG) pathway enrichment analysis based on SNPs used as instrumental variables in the Mendelian Randomization analysis using SNPnexus database(https://www.snpnexus.org/v4/, accessed on 25 September 2025).

In this study, we first analyzed the age-standardized disability-adjusted life years (DALY) rate of pediatric ASD (ages 0–14) worldwide in 2021 using the Global Burden of Disease (GBD) database. As shown in Figure 1A, the results indicated that although China’s DALY rate for pediatric ASD is relatively low compared to global standards, it remains notably higher than that of neighboring countries and territories in the Asia-Pacific region. This finding suggests a potential regional disparity in ASD burden that warrants further investigation. Additionally, the Global Burden of Disease Attributable to Ambient Particulate Matter Pollution for All Ages and Both Sexes in 2021 identified China as a prominent region significantly affected by ambient particulate matter pollution (Supplemental Figure S2). The convergence of elevated ASD burden and high ambient PM_2.5_ concentrations in China presents a compelling case for investigating potential causal relationships. Therefore, investigating the potential neurological impact of air pollution on pediatric ASD is both scientifically meaningful and of urgent public health importance. To explore this relationship further, we conducted Spearman correlation analysis between country/regional PM_2.5_ concentrations and age-standardized DALY rates for ASD across multiple nations and territories. As shown in Figure 1B, the results revealed a significant positive association (*p* < 0.01, R = 0.51), indicating a moderate to strong correlation between ambient PM_2.5_ levels and ASD-related disease burden at the population level. These ecological findings provide compelling preliminary evidence that air pollution may be associated with increased ASD severity risk at the population level, supporting the biological plausibility of our hypothesis. However, it is important to note that this ecological analysis cannot establish causality due to potential confounding factors such as healthcare accessibility, diagnostic practices, socioeconomic status, and other environmental exposures that may vary between countries. Nevertheless, these population-level associations provide important context and justification for more detailed mechanistic and epidemiological investigations into the relationship between air pollution exposure and autism spectrum disorders.

Subsequently, we performed Mendelian randomization analysis using GWAS data for air pollutants (PM_2.5_, PM_2.5_–PM_10_, PM_10_, and NO_x_) from the IEU OpenGWAS database and ASD severity risk data from the Psychiatric Genomics Consortium (Supplemental Table S3). The results indicated potential causal associations between PM_2.5_ and NOx exposure and ASD severity risk (Figure 1C). Additionally, increased PM_2.5_ levels were associated with elevated ASD severity risk (Figure 1D). Finally, we used the SNPnexus database to annotate SNPs to genes based on the instrumental variables employed in the Mendelian randomization analysis, followed by Gene Ontology (GO) molecular function enrichment and KEGG pathway enrichment analyses [28]. The GO molecular function enrichment highlighted glycosyltransferase activity, which is essential for numerous processes critical to brain development and function, and calcium:monoatomic cation antiporter activity, which plays important roles in synaptic transmission and neuronal development (Figure 1E). KEGG pathway analysis also emphasized the calcium signaling pathway and long-term potentiation, which are crucial for the development of complex social and communication skills as well as learning and memory processes [29,30]. These results suggest that PM_2.5_ exposure may adversely affect brain development, thereby increasing ASD severity risk.

### 3.2. PM_2.5_ Maybe the Predominant Air Pollutant Affecting ASD Symptom Severity

**Figure 2:**
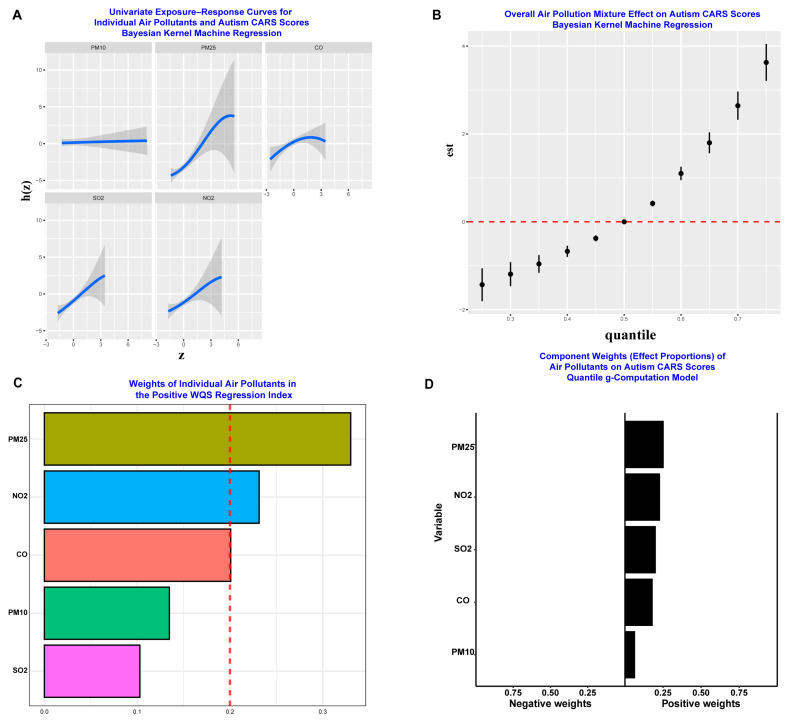
Association Between Exposure to Air Pollution Mixtures and Autism Severity (CARS Scores) Using Multiple Co-Exposure Statistical Models. (**A**) Univariate exposure-response curves generated by Bayesian Kernel Machine Regression (BKMR), illustrating the independent and potentially non-linear associations of individual air pollutants with Childhood Autism Rating Scale (CARS) scores. The solid blue line represents the estimated effect, with the shaded gray area denoting the 95% credible interval. (**B**) Overall effect of the combined air pollution mixture on CARS scores, as estimated by the BKMR model. (**C**) Weighted Quantile Sum (WQS) regression results, showing the estimated weights of individual pollutants within the mixture index. These weights reflect the relative contribution of each pollutant to the overall positive association with CARS scores, withPM_2.5_ contributing the most. (**D**) Component weights from the Quantile g-Computation model (qgcomp), indicating the proportional contribution of each air pollutant to the total mixture effect. All pollutants exhibit positive weights, suggesting a consistent association with higher CARS scores.

Community-level daily air pollutant data, including particulate matter (PM_10_ and PM_2.5_) and gaseous pollutants (CO, SO_2_, and NO_2_), were obtained from the China High Air Pollutants (CHAP) database following established protocols [31]. The CHAP database is recognized as a robust and reliable source of air pollution data for China and has been extensively utilized in epidemiological research. This community-level data ensures high spatial accuracy and representativeness of exposure assessment for our study population. The BKMR univariate dose–response curves (Figure 2A) revealed potential positive associations between all measured air pollutants (PM_10_, PM_2.5_, CO, SO_2_, and NO_2_) and ASD symptom severity, with the most pronounced effects observed at high concentration levels. These findings suggest that elevated exposure to any of these pollutants is associated with increased autism symptom severity. The BKMR overall effect plot (Figure 2B) demonstrated a distinctive non-linear, J-shaped relationship between the air pollutant mixture and ASD symptom severity. This pattern indicates a relatively stable baseline effect at lower exposure levels, followed by a statistically significant and increasingly strong positive association when the pollutant mixture reaches high concentrations. The J-shaped curve suggests potential threshold effects wherein pollutant impacts become markedly more pronounced beyond certain exposure levels. Additionally, the BKMR single-exposure effect analysis indicated that the overall health risk associated with the air pollutant mixture is primarily driven by PM_2.5_ and SO_2_ (Supplemental Figure S3), with PM _2.5_ showing the strongest individual contribution to the observed associations with ASD symptom severity. Collectively, the BKMR results provide compelling evidence that PM_2.5_ may be the predominant air pollutant affecting ASD symptom severity among the measured pollutants.

Subsequently, we conducted WQS and qgcomp analyses to validate whether PM_2.5_ is the predominant air pollutant affecting ASD symptom severity, providing complementary perspectives on mixture effects. In the WQS model, PM_2.5_ exhibited the largest weight, followed by NO_2_, with both weights substantially exceeding the conventional significance threshold of 0.2 (Figure 2C). This finding indicates that PM_2.5_ and NO_2_ are the most influential components driving the positive association between the air pollution mixture and ASD symptom severity. The WQS results corroborate the BKMR findings by confirming PM_2.5_’s predominant role to the observed health effects. In contrast, the qgcomp model analysis revealed a more nuanced picture of mixture effects (Figure 2D). Notably, this analysis demonstrated that no single pollutant serves as an overwhelmingly dominant driver of the mixture’s effect on autism symptoms. Instead, the adverse effect on CARS scores appears to result from the cumulative impact of the entire air pollution profile acting synergistically, with each component contributing relatively equally to the total effect. This finding suggests that neurotoxic effects may emerge from complex interactions among multiple pollutants rather than from the isolated action of individual components. The convergent evidence from these three complementary analytical approaches provides important insights into the nature of air pollution’s impact on ASD symptom severity. While BKMR and WQS analyses consistently identified PM_2.5_ as the potentially most neurotoxic individual component, the qgcomp results offer a broader perspective, indicating that the overall health risk may not stem from one or two specific pollutants alone, but rather from the cumulative and potentially synergistic impact of the entire pollution mixture. This methodological triangulation suggests that PM_2.5_ represents the most significant individual contributor among the measured pollutants. However, the complete health impact likely results from complex multi-pollutant interactions that cannot be fully captured by examining individual pollutants in isolation. These findings underscore the importance of considering air pollution as a complex environmental mixture when developing public health strategies and regulatory policies for protecting vulnerable populations, particularly children with neurodevelopmental disorders.

### 3.3. PM_2.5_ Exposure May Alter Gene Expression Related to Inflammation and Neuronal Development to Affect ASD Symptom Severity

**Figure 3:**
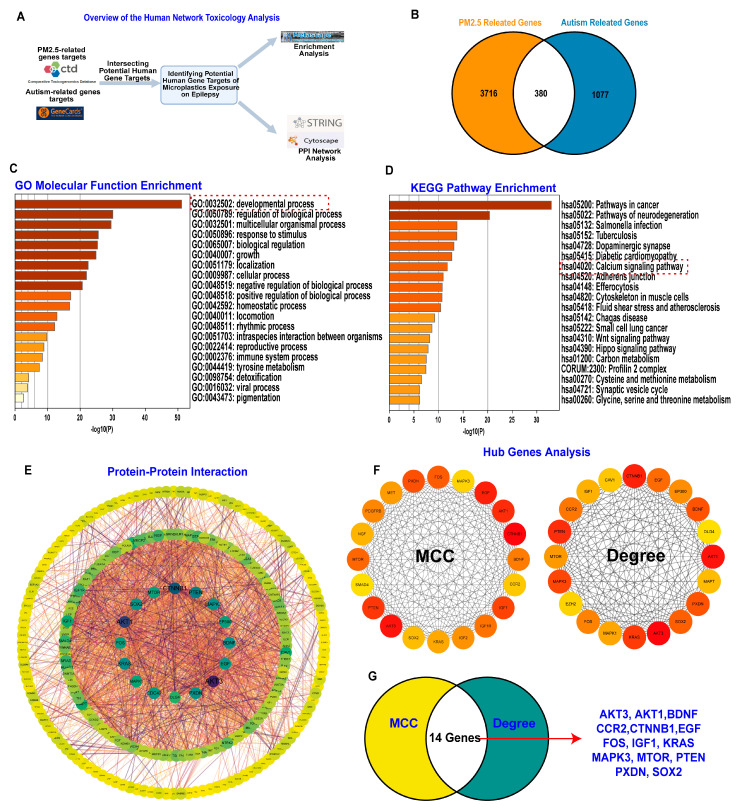
Human Network Toxicology Analysis of Potential Gene Targets Linking PM_2.5_ Exposure and Autism. (**A**) Flowchart outlining the methodological approach for the human network toxicology analysis. (**B**) Venn diagram showing the overlap between PM_2.5_-related genes and autism-related genes. (**C**) Bar plot presenting GO molecular function enrichment results for the intersecting genes. (**D**) Bar plot illustrating KEGG pathway enrichment results for the intersecting genes. (**E**) Visualization of the protein–protein interaction (PPI) network constructed from the intersecting gene set. (**F**) Hub gene analysis identifying top-ranked hub genes using two algorithms: Maximal Clique Centrality (MCC) and Degree. (**G**) Venn diagram displaying the 14 overlapping hub genes identified by both MCC and Degree algorithms: AKT3, AKT1, BDNF, CCR2, CTNNB1, EGF, FOS, IGF1, KRAS, MAPK3, mTOR, PTEN, PXDN, and SOX2.

Building on our previous research, we used human network toxicology analysis to identify target genes involved in the neuropathology of how PM_2.5_ exposure affects ASD symptoms [15,26] (Figure 3A). As shown in Figure 3B, the Venn diagram indicated that 360 overlapping genes participate in the neurological impact of PM_2.5_ exposure on ASD symptoms. We then conducted GO molecular function and KEGG pathway enrichment analyses based on these overlapping genes. The results highlighted neuronal developmental processes and calcium signaling pathways, which are consistent with our previous findings and further support the hypothesis that PM_2.5_ exposure may adversely affect brain development, thereby increasing ASD severity risk (Figure 3C,D). Furthermore, our integrated analysis of protein–protein interactions and hub genes pinpointed 14 genes as playing a probable key role in the neurological link between PM_2.5_ exposure and ASD.

**Figure 4:**
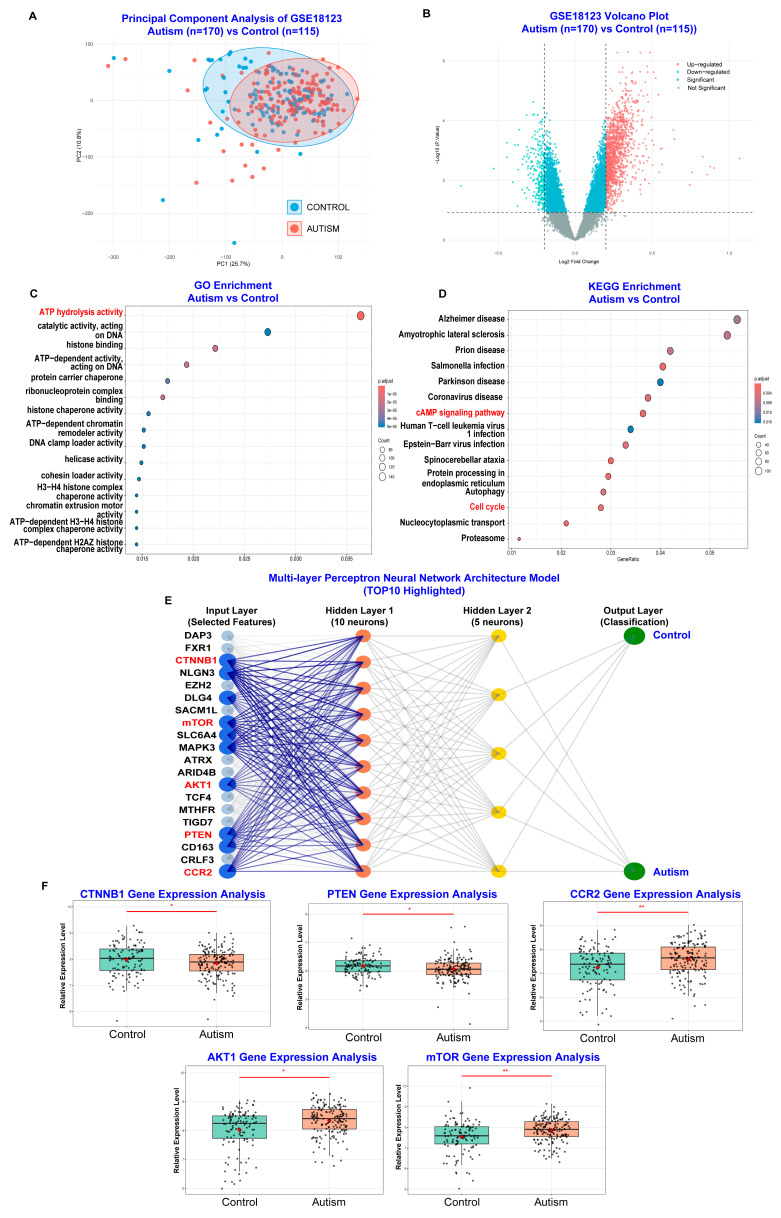
Bioinformatic and Machine Learning Analysis of Gene Expression in Autism Spectrum Disorder (ASD) Based on the GEO database. (**A**) Principal Component Analysis (PCA) of gene expression profiles from ASD patients (n = 170, red) and control subjects (n = 115, blue), demonstrating clear separation between the two groups. (**B**) Volcano plot depicting differentially expressed genes (DEGs) between ASD and control groups. Genes significantly upregulated in ASD are highlighted in red, while significantly downregulated genes are shown in blue. (**C**) GO enrichment analysis of DEGs, highlighting biological processes of the ATP hydrolysis in ASD. (**D**) KEGG pathway enrichment analysis of DEGs, indicating involvement of key pathways such as cAMP signaling and cell cycle regulation in ASD. (**E**) Architecture of the multi-layer perceptron neural network model for classifying ASD and control samples. The input layer includes selected gene features, with the top 10 most influential genes (including CTNNB1, PTEN, CCR2, AKT1, and mTOR) highlighted. (**F**) Boxplots comparing the relative expression levels of five key genes (CTNNB1, PTEN, CCR2, AKT1, and mTOR) between ASD and control groups. Each dot represents an individual sample, confirming significant differential expression of these genes in ASD. * *p* < 0.05, ** *p* < 0.01.

Subsequently, we conducted bioinformatic and machine learning analyses of GSE18123 from the Gene Expression Omnibus (GEO) database (Figure 4A,B), which included gene expression data from peripheral blood of ASD patients (n = 170) and healthy control subjects (n = 115). This independent dataset was used to identify and validate the hub genes obtained from our human network toxicology analysis and assess their roles in ASD pathophysiology [32]. As shown in Figure 4C,D, GO molecular function enrichment analysis highlighted ATP hydrolysis activity, also known as ATPases, which is fundamental for calcium signaling pathways and brain development [33]. Meanwhile, KEGG pathway enrichment analysis highlighted the cAMP signaling pathway and cell cycle, which are important for synaptic plasticity and neuronal development [34].Additionally, as shown in Figure 4E, using a multi-layer perceptron neural network model architecture, we identified the top 10 genes involved in the pathophysiology of ASD. 5 genes were selected as overlapping genes involved in both the neurological impact of PM_2.5_ exposure on ASD symptoms and the pathophysiology of ASD (CTNNB1, PTEN, CCR2, AKT1, and mTOR). And gene expression analysis revealed that the pathophysiology of ASD involves alterations in CCR2 gene expression, a biomarker for inflammatory response, and alterations in CTNNB1, PTEN, AKT1, and mTOR gene expression, biomarkers for neuronal development (Figure 4F).

**Figure 5:**
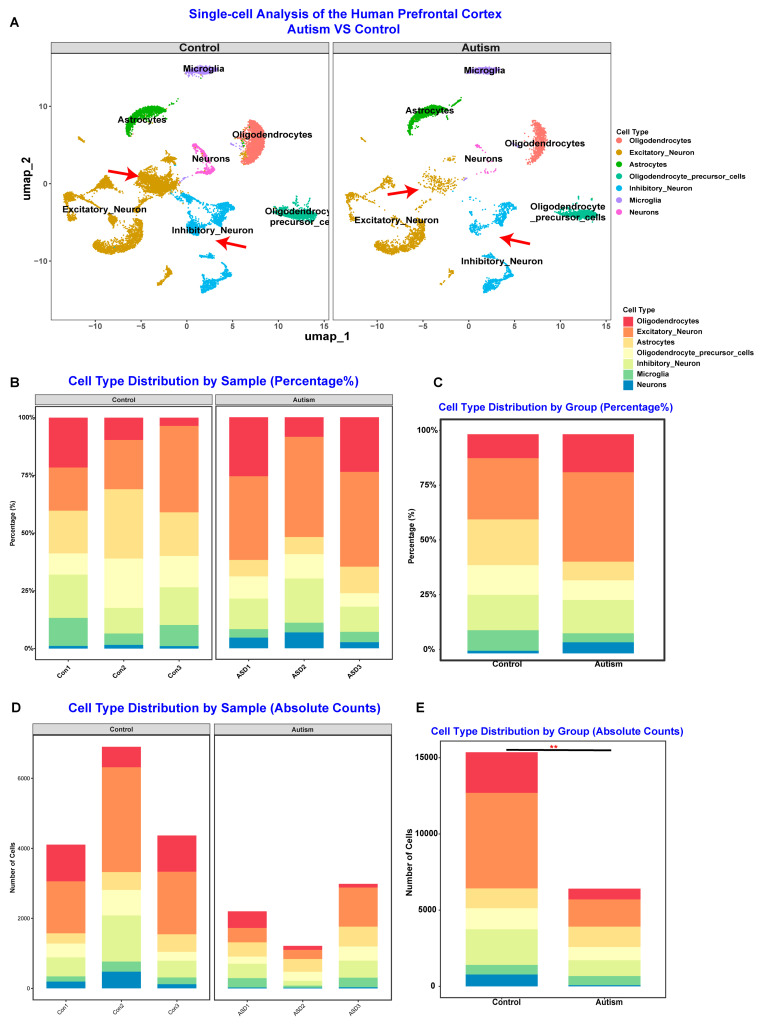
Single-cell RNA Sequencing Analysis of the Prefrontal Cortex from Individuals with Autism Spectrum Disorder (ASD) Based on the DISCO Database. (**A**) Uniform Manifold Approximation and Projection (UMAP) plots visualizing cell clusters in the prefrontal cortex from control (**left**) and ASD (**right**) samples. Each point represents a single cell, colored by cell type. Red arrows highlight significant differences in excitatory and inhibitory neuron clusters between the two groups. (**B**) Stacked bar charts showing the relative percentage of each cell type across individual control (Con1–Con3) and ASD (ASD1–ASD3) samples. (**C**) Stacked bar chart comparing the overall cell type distribution as a percentage between the aggregated control and ASD groups. (**D**) Stacked bar charts showing the absolute number of cells for each cell type recovered from individual control and ASD samples. (**E**) Stacked bar chart comparing the total absolute counts of each cell type between the aggregated control and ASD groups. ** *p* < 0.01.

**Figure 6:**
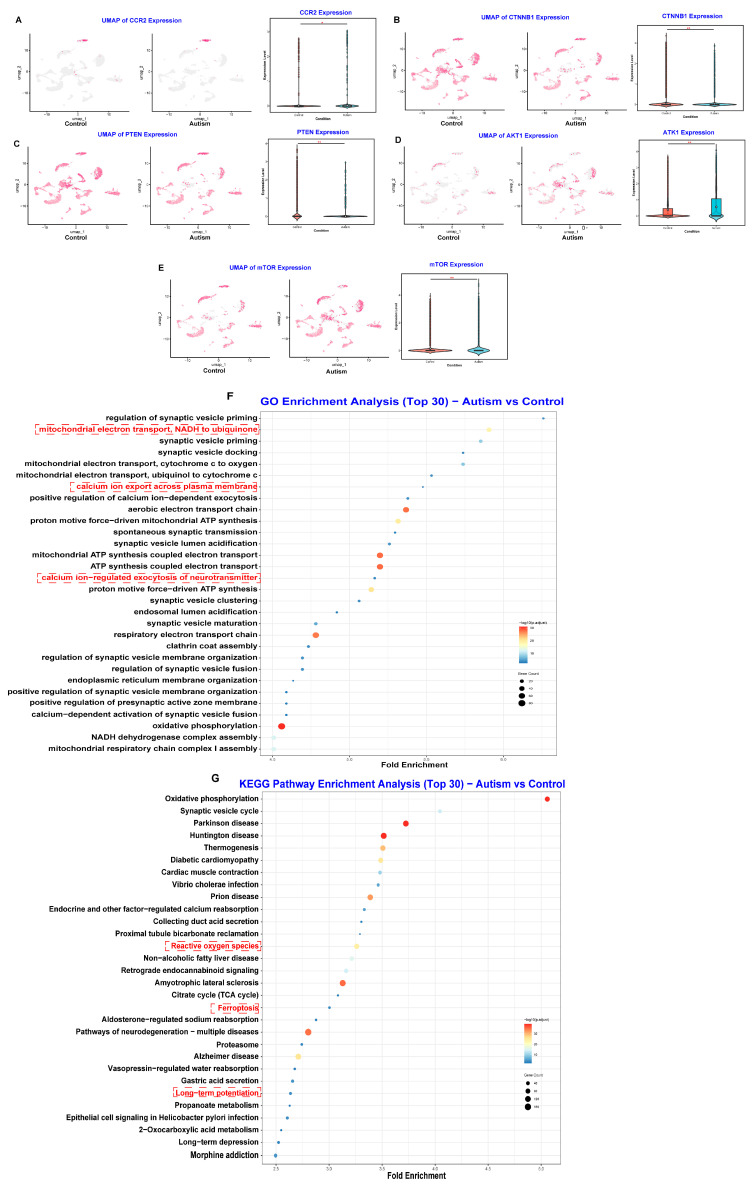
Single-Cell Expression of Key Genes and Functional Enrichment Analysis in the Prefrontal Cortex of ASD. (**A**–**E**) Visualization of the expression of five key genes (CCR2, CTNNB1, PTEN, AKT1, and mTOR) at the single-cell level. For each gene, the UMAP plots (**left**) display the spatial distribution of gene expression across all cell types in control and ASD samples (higher expression shown with more intense red). The violin plots (**right**) compare the overall expression levels of each gene between control (blue) and ASD (red) groups. (**F**) GO enrichment analysis of differentially expressed genes between ASD and control samples (Top 30 terms). (**G**) KEGG pathway enrichment analysis of differentially expressed genes between ASD and control samples (Top 30 pathways). * *p* < 0.05, ** *p* < 0.01.

Finally, we conducted single-cell RNA sequencing (scRNA-seq) analysis of prefrontal cortex tissue from individuals with ASD using the DISCO database (n = 3) as an external validation dataset to further confirm whether these five hub genes participate in ASD pathophysiology at the level of the primary affected organ system [35]. As shown in Supplemental Figure S4–S8 and Figure 5A, the UMAP comparison between the prefrontal cortex from individuals with ASD and healthy controls indicated that the pathophysiology of ASD involves the imbalance of excitatory and inhibitory neurons. Additionally, Figure 5B,C further indicated that the pathophysiology of ASD involves not only significant neuron loss but also changes in the relative levels of neurons, particularly for excitatory and inhibitory neurons (Figure 5B–E). Moreover, gene expression analyses support that altered gene expression of CTNNB1, PTEN, CCR2, AKT1, and mTOR is involved in the pathophysiology of ASD (Figure 6A–D). GO molecular function and KEGG pathway enrichment analyses highlighted the key roles of calcium signaling pathways, ferroptosis, and long-term potentiation (Figure 6E,F). Overall, these results indicate that PM_2.5_ exposure may alter gene expression related to inflammation and neuronal development to affect ASD symptom severity.

### 3.4. PM_2.5_ Exposure May Exacerbate the VPA-Induced ASD Like Behaviors in Mice

**Figure 7:**
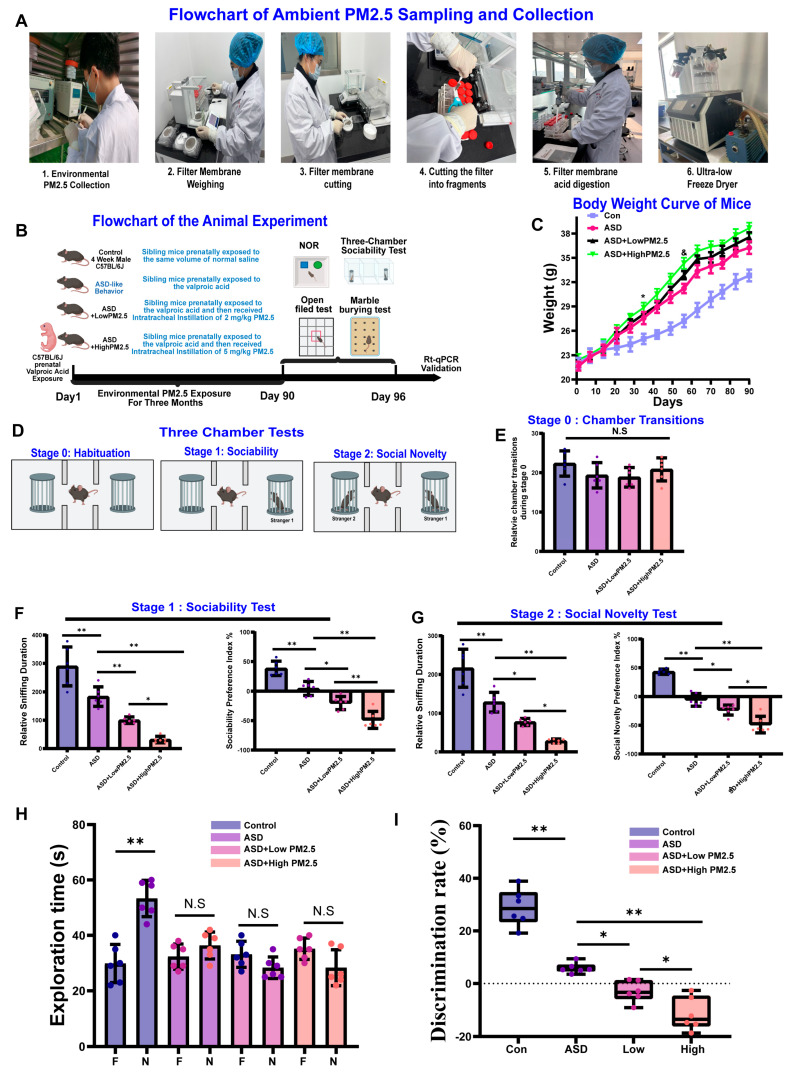
Effects of sustained environmental Ambient PM_2.5_ Exposure on Valproic Acid (VPA)-Induced Autism-Like Behaviors in Mice. (**A**) Flowchart illustrating the main steps for ambient PM_2.5_ sampling and collection. (**B**) Schematic of the animal experimental design, including prenatal VPA exposure and subsequent PM_2.5_ treatments. (**C**) Body weight curves of mice from four experimental groups over the 90-day exposure period. * indicate days on which body weight significantly differed from the control group (*p* < 0.05); & denote days with a significant difference between the ASD + Low PM_2.5_ and ASD + High PM_2.5_ groups (*p* < 0.05). (**D**) Diagram of the three-chamber social interaction test, showing the stages of habituation, sociability, and social novelty. (**E**) Results of chamber transitions during Stage 0. (**F**) Results from Stage 1 (Sociability Test), including relative sniffing duration (**left**) and sociability preference index (**right**) for each group. (**G**) Results from Stage 2 (Social Novelty Test), including relative sniffing duration (**left**) and social novelty preference index (**right**) for each group. (**H**) Novel Object Recognition (NOR) test results, showing exploration time for familiar (**I**) and novel (N) objects in each group. (**I**) Boxplot of the discrimination rate from the NOR test, reflecting cognitive and memory performance across the four groups. * *p* < 0.05, ** *p* < 0.01; N.S., not significant.

To validate the neurological impact of PM_2.5_ exposure on ASD and its potential target genes, 4-week-old male offspring exhibiting ASD-like behaviors following prenatal VPA exposure were selected as the experimental ASD model to approximate the pediatric population (Figure 7B). As shown in Figure 7C, the body weight curve indicated that long-term low-level environmental PM_2.5_ exposure may increase the body weight of ASD mice, and high-level PM_2.5_ exposure further exacerbated these adverse effects, possibly due to PM_2.5_ exposure inducing inflammation and disturbing lipid metabolism [36]. To assess the neurological impact of PM_2.5_ exposure on social deficits in ASD mice, a three-chamber social test was conducted based on previous studies (Figure 7D) [37]. In Stage 0, we quantified chamber transitions for all groups to ensure that observed behavioral changes in social interaction were not attributable to general locomotor activity deficits. As shown in Figure 7E, no significant differences in chamber transitions were observed among all groups (*p* > 0.05), indicating that locomotor function remained intact across all experimental conditions. As shown in Figure 7F, in the sociability test period of the three-chamber social test, compared to the control group, ASD mice presented with significantly reduced sociability (*p* < 0.05), while long-term low-level environmental PM_2.5_ exposure significantly exacerbated these adverse effects (*p* < 0.05). Additionally, long-term high-level environmental PM_2.5_ exposure further significantly aggravated the adverse effects (*p* < 0.05), and similar changes were observed in the impaired social novelty preference (Figure 7G).

**Figure 8:**
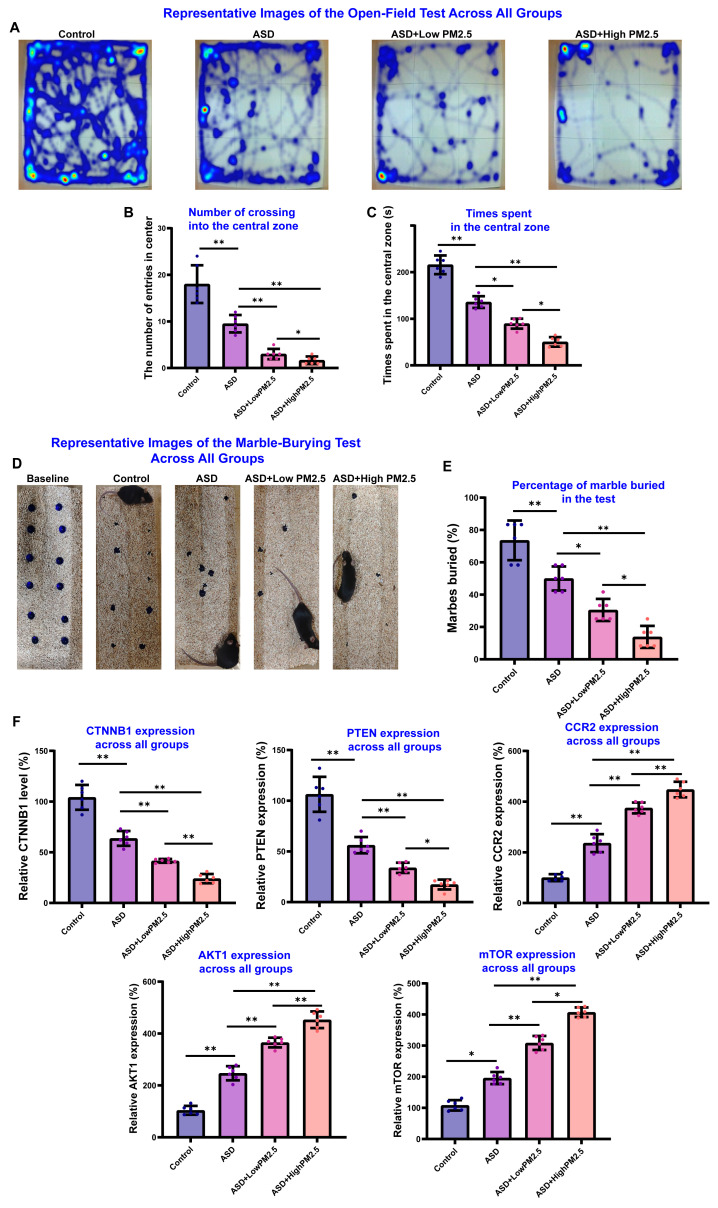
Effects of sustained environmentalAmbient PM_2.5_ Exposure on Valproic Acid (VPA)-Induced Autism-Like Behaviors in Mice. (**A**) Representative heatmaps from the open-field test, illustrating locomotor activity and exploration patterns across all experimental groups. (**B**) Quantification of the number of entries into the central zone of the open-field arena. (**C**) Quantification of the total time spent in the central zone during the open-field test. (**D**) Representative images from the marble-burying test across all groups. (**E**) Quantification of the percentage of marbles buried by mice in each group during the test. (**F**) Relative mRNA expression levels of five selected genes (CTNNB1, PTEN, CCR2, AKT1, and mTOR) in the prefrontal cortex of mouse brain tissue across all groups, as measured by RT-qPCR. * *p* < 0.05, ** *p* < 0.01.

To assess the neurological impact of PM_2.5_ exposure on cognitive deficits in ASD mice, the novel object recognition test was conducted. As shown in Figure 7H,I, there was a significant difference between time spent with familiar and novel objects in the control group, indicating that they could distinguish familiar objects from new ones (*p* < 0.05). However, the ASD mice and long-term low-level and high-level environmental PM_2.5_ exposure groups showed no significant difference between time spent with familiar and novel objects (*p* > 0.05), indicating impairment in recognition memory in these groups. Additionally, the discrimination rate of the novel object recognition test indicated that PM_2.5_ exposure may exacerbate VPA-induced cognitive deficits in mice. To further investigate the impact of PM_2.5_ exposure on the neurological phenotype of ASD mice, we assessed anxiety-like and repetitive behaviors using the open-field test and the marble-burying test. Our results revealed that PM_2.5_ exposure significantly exacerbated anxiety-related behaviors in ASD mice, mirroring the detrimental effects observed in social and cognitive domains. In the open-field test, representative tracking plots (Figure 8A) visually demonstrate that ASD mice exhibit greater anxiety-like behaviors compared to control mice [26]. This behavior was markedly intensified following exposure to PM_2.5_. Quantification of this behavior confirmed that both the number of entries into the central zone (Figure 8B) and the total time spent in the central zone (Figure 8C) were significantly reduced in ASD mice. This reduction was further amplified in a dose-dependent manner by both low and high concentrations of PM_2.5_, indicating a heightened state of anxiety. Similarly, in the marble-burying test, which evaluates repetitive and anxiety-driven behaviors, ASD mice buried a significantly lower percentage of marbles compared to the control group (Figure 8D,E). This deficit was significantly worsened by PM_2.5_ exposure, again in a dose-dependent fashion, suggesting an exacerbation of ASD-related behavioral abnormalities. Finally, to probe the underlying molecular mechanisms, we used RT-qPCR to analyze the expression of genes associated with neuronal development and neuroinflammation (Figure 8F). We found that the expression levels of CTNNB1 and PTEN, genes crucial for neuronal development and synaptic function, were downregulated in ASD mice and further suppressed by PM_2.5_ exposure. Conversely, the expression of genes linked to inflammatory signaling and cell metabolism, including CCR2, AKT1, and mTOR, was significantly upregulated in the ASD group and was further elevated by PM_2.5_ in a dose-dependent manner. Collectively, these findings suggest that exposure to PM_2.5_ aggravates anxiety-like behaviors in ASD mice. This behavioral exacerbation is correlated with the dysregulation of key genes involved in both neuronal development and inflammatory pathways, providing a potential molecular basis for the observed effects.

## 4. Discussion

With the rapid development of industry, increasing research has recently revealed a troubling link between air pollution and an increased risk of various pediatric neurological disorders [38]. The developing brain of children is uniquely vulnerable to the toxic effects of pollutants, which can have long-lasting consequences for a child’s cognitive and behavioral health, prompting urgent calls for further investigation into this pervasive environmental threat [39]. As one of the most common neurodevelopmental conditions in children, ASD involves primary challenges in social interaction and communication, which can co-occur with cognitive difficulties and anxiety [40]. While genetic factors are known to play a significant role, the rising prevalence of ASD suggests that environmental factors are also key contributors. Numerous epidemiological studies have identified air pollution, particularly PM_2.5_, as a key environmental risk factor for ASD. This research establishes a clear link between exposure during prenatal and early childhood periods and a higher probability of diagnosis [41]. However, investigations regarding whether sustained environmental PM_2.5_ exposure may exacerbate symptoms in children with ASD, as well as its potential mechanisms, remain limited. Accordingly, we aimed not only to explore the link between long-term PM_2.5_ exposure and the severity of ASD symptoms but also to investigate the fundamental mechanisms responsible for this effect.

In this study, we initially combined the GBD database and IQAir database to provide preliminary evidence that air pollution may be associated with increased ASD severity risk at the population level. Combined Mendelian randomization analysis and enrichment analyses highlighted the key roles of glycosyltransferase activity, calcium signaling pathway, and long-term potentiation in the neurological impact of PM_2.5_ on ASD. Glycosyltransferase activity is emerging as a significant factor in the neurobiology of ASD [42]. This enzymatic activity is crucial for glycosylation, a process that attaches sugar chains to proteins and lipids, which is vital for the proper function and development of the central nervous system. Long-term potentiation (LTP) is a persistent strengthening of synapses based on recent patterns of activity and is widely considered to be a primary cellular mechanism underlying learning and memory [43]. The calcium signaling pathway is at the heart of the induction of many forms of LTP, particularly in the hippocampus, a brain region critical for memory formation [44]. These results support that PM_2.5_ exposure may adversely affect brain development, thereby increasing ASD severity risk.

Subsequently, we conducted a comprehensive 3-year prospective observational cohort study involving 321 children diagnosed with autism spectrum disorders to rigorously validate the effects of ambient air pollution exposure on ASD symptom severity and progression. This longitudinal design allowed us to capture temporal variations in both pollutant exposure and clinical manifestations, providing robust evidence for causal inference. Employing a sophisticated multi-model analytical approach that integrated Bayesian Kernel Machine Regression (BKMR), Weighted Quantile Sum (WQS) regression, and Quantile g-Computation (qcomp) models, our comprehensive analyses consistently highlighted that PM_2.5_ exposure significantly influences ASD symptom severity, with particularly pronounced effects observed at elevated concentration levels. This multi-model convergence strengthens the reliability of our findings by accounting for different statistical assumptions and methodological approaches to mixture analysis. Notably, an intriguing pattern emerged in our BKMR analysis: we observed a distinct leveling-off or plateau effect in the dose–response curve for PM_2.5_ at extremely high concentrations. This nonlinear relationship warrants careful interpretation, as it is likely attributable to statistical limitations rather than genuine biological saturation effects. Specifically, very few study participants were exposed to these extreme pollutant levels in our cohort, with the majority of exposure data concentrated around mean or median values. This distribution pattern may have introduced a statistical artifact due to sparse data in the high-exposure tail, potentially masking the true dose–response relationship at these extreme concentrations rather than representing an authentic biological threshold or protective mechanism. Overall, this comprehensive investigation systematically analyzed the effects of a complex 5-pollutant air pollution mixture on ASD symptom manifestation using multiple advanced statistical modeling approaches. The results demonstrate remarkable consistency across methodologies, uniformly showing that the pollutant mixture is significantly associated with increased risk and severity of ASD symptoms. Most critically, both the BKMR and WQS models consistently and strongly identified PM_2.5_ as the predominant risk contributor within the mixture, establishing it as the primary driver of neurodevelopmental impacts. Complementing these findings, the quantile g-computation analysis revealed that the observed health risks represent a cumulative effect, wherein all components of the pollution mixture contribute additively to the overall neurodevelopmental burden. This finding underscores the importance of considering pollutant interactions and mixture effects rather than focusing solely on individual components. Collectively, these convergent findings emphasize the critical importance of implementing comprehensive air quality control strategies, with particular emphasis on PM_2.5_ reduction, to protect children’s neurodevelopmental health and potentially mitigate the severity of autism spectrum disorder symptoms. These results provide compelling evidence for public health policies aimed at reducing ambient air pollution exposure, especially in vulnerable pediatric populations. Human network analyses were also conducted to further investigate the potential gene targets underlying the neurological impact of PM_2.5_ exposure on ASD symptoms, building upon our previous comprehensive studies. These systematic analyses highlighted 14 genes as potentially key regulatory nodes in the pathophysiological networks linking environmental particulate matter exposure to autism spectrum disorder manifestations.

To enhance the precision and reliability of our gene identification approach, we employed a sophisticated multi-layer perceptron neural network model architecture integrated with gene expression profiles derived from peripheral blood samples of children diagnosed with ASD. Through this advanced computational framework, we successfully identified five critical overlapping genes: CTNNB1, PTEN, CCR2, AKT1, and mTOR. These genes represent crucial molecular intersections involved in both the neurological consequences of PM_2.5_ exposure on ASD symptomatology and the fundamental pathophysiological mechanisms underlying autism spectrum disorders. Single-cell RNA sequencing analysis of prefrontal cortex tissue samples from individuals with ASD provided additional validation and mechanistic insights, confirming differential expression patterns of these identified genes. This high-resolution cellular analysis particularly highlighted the pivotal roles of three interconnected biological processes: calcium signaling pathways, ferroptosis, and long-term potentiation mechanisms. Notably, the nexus of these three critical processes lies in the multifaceted and central role of calcium signaling in neuronal function and dysfunction. Calcium ions serve as essential second messengers in synaptic transmission, neuroplasticity, and cellular homeostasis. However, when dysregulated, excessive and sustained calcium influx—often referred to as calcium overload or calcium toxicity—can become a harbinger of neuronal cell death and synaptic dysfunction. This pathological calcium dysregulation can trigger cascading events including mitochondrial dysfunction, oxidative stress, activation of proteases and lipases, and ultimately, the initiation of various cell death pathways including ferroptosis, thereby creating a destructive cycle that compromises neural circuit integrity and function in autism spectrum disorders [15,16,18,19]. This calcium overload can trigger several interconnected pathways that actively promote ferroptosis, creating a cascade of cellular dysfunction. Elevated intracellular calcium concentrations can activate calcium-dependent enzymes, including phospholipase A2 and NADPH oxidases, which generate reactive oxygen species (ROS) and directly contribute to the lipid peroxidation processes that are hallmarks of ferroptotic cell death [45,46]. The accumulation of lipid peroxides, particularly those containing polyunsaturated fatty acids, creates a toxic cellular environment that overwhelms antioxidant defense systems and accelerates neuronal damage. Furthermore, calcium dysregulation profoundly impairs mitochondrial function, representing another critical factor in the progression of ferroptosis. Excessive calcium uptake by mitochondria disrupts the electron transport chain, reduces ATP production, and enhances mitochondrial ROS generation, thereby creating a self-perpetuating cycle of oxidative damage and iron-dependent cell death [45,46]. This mitochondrial dysfunction not only compromises cellular energy metabolism but also impairs the cell’s ability to maintain iron homeostasis and antioxidant capacity. Intriguingly, the single-cell RNA sequencing analysis of prefrontal cortex tissue from individuals with ASD also revealed significant cellular composition abnormalities, particularly highlighting a pronounced imbalance between excitatory and inhibitory neuronal populations. This excitatory-inhibitory (E/I) imbalance represents a fundamental disruption in neural circuit dynamics that has emerged as a core pathophysiological feature of autism spectrum disorders. The primary contributor to this critical imbalance appears to be a systematic weakening of the brain’s inhibitory “braking” system, which predominantly involves the GABAergic neurotransmitter system. GABA (gamma-aminobutyric acid) serves as the brain’s primary inhibitory neurotransmitter, and its dysfunction can lead to hyperexcitability and altered neural oscillations. Compelling evidence from post-mortem neuropathological studies of individuals with autism has consistently demonstrated a reduced number of specific subtypes of GABAergic inhibitory interneurons, particularly parvalbumin-positive fast-spiking interneurons and somatostatin-positive interneurons, which play crucial roles in maintaining proper excitatory-inhibitory balance and supporting gamma oscillations essential for cognitive function and sensory processing [47]. While inhibitory systems are often weakened, excitatory systems can become overactive, on the one hand, many genes strongly associated with ASD, such as those in the SHANK and Neurexin-Neuroligin families, are critical for building and maintaining excitatory synapses [48]. On the other hand, mutations can lead to synapses that are either too strong, too numerous, or improperly regulated, causing excessive “go” signals. Additionally, the function and number of glutamate receptors (like NMDA and AMPA receptors) can be altered, making neurons hyperexcitable and overly sensitive to incoming signals [49]. In summary, the imbalance of excitatory and inhibitory neurons can make brain difficult to extract meaningful information from social interactions as the processing of complex social cues requires incredibly fast and precise coordination between excitatory and inhibitory signals.

Finally, using 4-week-old male offspring exhibiting ASD-like behaviors following prenatal VPA exposure as the experimental ASD model to approximate the pediatric population, we validated the neurological impact of PM_2.5_ exposure on ASD and the PM_2.5_ exposure-induced alterations in gene expression of CTNNB1, PTEN, CCR2, AKT1, and mTOR. Genes critical for brain development and implicated in ASD are susceptible to epigenetic modifications—changes that control their expression without altering DNA. Mounting evidence suggests that air pollution can be a trigger for these exact modifications. CTNNB1 is a gene that codes for β-catenin, a protein vital for cell-to-cell adhesion and gene expression via the Wnt signaling pathway; it is critical for synapse development and stability [50]. Mutations in CTNNB1 are linked to a specific neurodevelopmental syndrome that includes intellectual disability and features of autism [51]. Disruption of this pathway alters synapse formation and function, contributing to the core symptoms of ASD [51]. Additionally, our results indicated that the PTEN/AKT/mTOR pathway is a central hub, and PM_2.5_ exposure may further decrease the expression of CTNNB1 and PTEN, accompanied by increases in CCR2, AKT1, and mTOR in ASD mice. Our results indicated that PTEN may act as a brake, promoting ferroptosis and keeping cell growth in check, while AKT and mTOR are accelerators, promoting survival, growth, and the protein synthesis needed for LTP [52,53]. Previous studies indicated that PTEN aims to prevent cells from growing and dividing too rapidly. When PTEN is functioning correctly, it helps maintain cellular balance. However, mutations in the PTEN gene can impair its ability to function, leading to uncontrolled activation of the PI3K/AKT/mTOR pathway [52,53]. Overactivation of the AKT/mTOR pathway is a central theme in ASD, which leads to the overproduction of proteins at the synapse, creating “sticky” and inflexible connections that cannot undergo LTP properly. This is thought to underlie some of the cognitive rigidity and learning difficulties seen in ASD. AKT1 is a crucial enzyme at the heart of the PI3K/AKT/mTOR signaling pathway, influencing a wide array of downstream targets. It is heavily involved in promoting cell survival and blocking programmed cell death such as ferroptosis, and previous studies indicated that AKT1 can phosphorylate and inhibit another protein called GSK3β. GSK3β normally promotes the degradation of NRF2. Therefore, by inhibiting GSK3β, AKT1 ensures that Nrf2 remains stable and active, allowing it to continue its protective, antioxidant functions to inhibit ferroptosis [54]. mTOR is a major downstream target of AKT1. When activated, mTOR boosts cell growth by promoting protein synthesis and limiting the cellular recycling process known as autophagy [55]. CCR2, a receptor on immune cells that directs them to sites of inflammation, connects to the neuro-immune axis of ASD. There is strong evidence for increased inflammation in the brains of many individuals with ASD. CCR2 and its signaling partner CCL2 are often elevated, indicating chronic neuroinflammation. This inflammatory environment is highly disruptive to brain function and represents a critical pathological mechanism in autism spectrum disorders. Neuroinflammation acts as a potent inhibitor of long-term potentiation (LTP), fundamentally compromising the brain’s capacity for synaptic strengthening and neural adaptation. The inflammatory molecules released by activated immune cells, including pro-inflammatory cytokines such as IL-1β, TNF-α, and IL-6, can directly interfere with synaptic plasticity mechanisms, systematically impairing learning and memory circuits and providing a clear mechanistic link between immune system dysfunction and the cognitive symptoms characteristic of ASD [56,57]. More critically, the neuroinflammation driven by this immune dysregulation serves as a direct upstream activator of the PTEN/AKT/mTOR signaling pathway, creating a synergistic and increasingly destructive pathological cycle that is now recognized as a core mechanism underlying a significant subset of ASD cases. This hyperactivated mTOR pathway leads to excessive protein synthesis, aberrant dendritic spine morphology, and disrupted synaptic homeostasis, while simultaneously promoting further inflammatory responses that perpetuate the cycle [58,59]. Overall, this chronically overactive signaling cascade, combined with dysregulated calcium signaling and altered excitatory–inhibitory balance, fundamentally disrupts LTP induction and maintenance, making it increasingly difficult for neural circuits to adapt, learn, and form appropriate connections during critical developmental windows. Concurrently, persistent factors such as neuroinflammation, oxidative stress, and microglial activation create a toxic cellular environment that progressively damages neuronal function, compromises synaptic integrity, and ultimately contributes to the complex behavioral and cognitive phenotypes observed in autism spectrum disorders.

## 5. Limitations

Several limitations of this study should be acknowledged. First, our findings are derived from a pilot observational cohort at a single location with a limited number of participants, which may constrain the statistical power of our analyses and the generalizability of our conclusions to populations with different genetic backgrounds, dietary habits, or socioeconomic statuses. Additionally, this analysis may not have adequately adjusted for key confounders such as parental socioeconomic status, comorbid conditions, or medication use, potentially resulting in residual confounding that could distort the observed associations. A larger, multi-center cohort study is necessary to validate our findings and enhance their broader applicability. Second, this study examined PM_2.5_ as a bulk mixture without analyzing its specific chemical composition. PM_2.5_ is a heterogeneous mixture comprising heavy metals, polycyclic aromatic hydrocarbons (PAHs), nitrates, sulfates, and potentially endotoxins (lipopolysaccharides), with composition varying significantly by geographic location, season, and emission source. Without component-specific analysis, including endotoxin quantification, identifying which constituents are responsible for the observed neurological effects remains challenging, thereby limiting our ability to pinpoint the most harmful elements. The potential contribution of endotoxin contamination to the observed inflammatory and neurotoxic effects cannot be ruled out and warrants specific investigation. Future studies incorporating comprehensive chemical characterization (LC/MS and GC/MS) and biological contaminant analysis (endotoxin assays) are essential to identify the specific toxicological components driving PM_2.5_ induced neurotoxicity in ASD models. Third, our findings may be confounded by co-exposure to other airborne pollutants, including SO_2_, NO_2_, and ozone. These pollutants frequently coexist with PM_2.5_ in urban environments and may exert independent or synergistic effects on neurodevelopment. Although our statistical models attempted to account for multiple pollutants, fully disentangling the specific health impacts of PM_2.5_ from the complex urban exposome remains challenging. Fourth, intratracheal instillation of PM_2.5_ may induce inflammatory responses in mice that could influence our results. While a sample size of n = 6 per group is generally adequate for detecting large effect sizes with well-controlled variability in behavioral studies, larger cohorts would strengthen statistical power for identifying more subtle or heterogeneous effects. Future animal experiments employing expanded sample sizes are recommended to corroborate our preliminary observations. Additionally, although we identified potential molecular pathways and validated changes in key gene expression, the complete molecular cascade linking PM_2.5_ exposure to ASD symptom exacerbation remains incompletely elucidated. Our research establishes strong associations and highlights critical genes (CTNNB1, PTEN, CCR2, AKT1, mTOR); however, the precise upstream triggers and downstream effector mechanisms require further investigation. Future studies employing techniques such as targeted genetic knockouts or pharmacological pathway inhibitors are needed to establish causal relationships for these genes. Finally, our reliance on a VPA-induced murine model, while widely accepted in the field, has inherent limitations. This model may not fully recapitulate the complex genetic and etiological heterogeneity of human ASD. Furthermore, intratracheal instillation of PM_2.5_, while ensuring precise dosage control, does not perfectly replicate the chronic, low-dose nature of natural inhalation exposure. This methodological difference could affect particle distribution, clearance kinetics, and ultimate biological impact, potentially limiting the direct translatability of our findings to real-world human exposure scenarios.

## 6. Conclusions

In summary, our multi-faceted study provides compelling evidence that long-term exposure to ambient PM_2.5_ exacerbates the symptoms of ASD. Through a comprehensive approach that integrates global burden of disease data, a regional observational cohort study, and in-depth animal experiments, we have demonstrated a significant association between PM_2.5_ levels and increased ASD severity. Our findings identify PM_2.5_ as a primary environmental determinant influencing ASD symptom manifestation. Furthermore, we have elucidated potential underlying molecular mechanisms, highlighting that PM_2.5_ exposure disrupts neurodevelopmental and inflammatory pathways. Specifically, we identified CTNNB1, PTEN, CCR2, AKT1, and mTOR as core target genes mediating these neurological impacts. These results not only advance our mechanistic understanding of how air pollution impacts neurodevelopmental disorders but also underscore the urgent need for public health policies aimed at reducing air pollution to protect vulnerable populations, especially children with ASD.

## Data Availability

Additional data will be made available upon reasonable request to the corresponding author.

## Supplementary Materials

The following supporting information can be downloaded at: https://www.mdpi.com/article/10.3390/toxics13110922/s1, File S1: Detailed information of other related information involved in this study; Figure S1: The flowchart of observational cohort in this study; Figure S2: The flowchart of observational cohort in this study; Figure S3: The BKMR single-exposure effect plot; Figure S4: Data Quality Control Before and After Filtering in scRNA-seq Analysis; Figure S5: Clustering Tree for scRNA-seq Analysis; Figure S6: UMAP Clustering at Different Resolutions; Figure S7: Heatmap of genes Markers Across All Cluster; Figure S8: Dotplot of Genes Markers Across All Cell Types; Table S1: The World Health Organization’s (WHO) new World Standard Population; Table S2: The basic characteristics of patients diagnosed with ASD involving in this study; Table S3: Detailed information of GWAS data used in the study; Table S4: Detailed information of primer sequences utilized in this study; References [60,61,62,63,64,65,66,67,68,69,70,71,72,73] are cited in Supplementary Materials.

## Correction Statement

This article has been republished with a minor correction to the copyright information/Acknowledgments/Author Contributions/Institutional Review Board Statement/Informed Consent Statement/Data Availability Statement/Conflicts of Interest Statement. This change does not affect the scientific content of the article.
