# Supplementary material for: Investigating the Effects of Long-Term Fine Particulate Matter Exposure on Autism Spectrum Disorder Severity: Evidence from Multiple Analytical Approaches
Source: Toxics. 2025 Oct 28;13(11):922. doi: 10.3390/toxics13110922 (PMC12656233; doi:10.3390/toxics13110922)
Supplement: Supplementary file 1 [file toxics-13-00922-s001.zip › toxics-3923160-supplementary.pdf]

Supplemental Information Section

Supplemental Information 1

Childhood Autism Rating Scale, Second Edition (CARS)

Directions: For each category, rate the behaviors relevant to each item of the scale. For each item, circle the number which corresponds to the statement that best describes your child. You may indicate the child is between two descriptions by using ratings of 1, 2, 3, 4. Abbreviated rating criteria are provided for each scale.

Category I. Relating to People:

Rating 1: No evidence of difficulty or abnormality in relating to people. The child's behavior is appropriate for his or her age. Some shyness, fussiness of annoyance at being told what to do may be observed, but not to an atypical degree

Rating 2: Mildly abnormal relationships. The child may avoid looking the adult in the eye, avoid the adult, or become fussy if interaction is forced, be excessively shy, not be as responsive to the adult as typical, or cling to parents somewhat more than most children of the same age.

Rating 3: Moderately abnormal relationships. The child shows aloofness(seems unaware of adults) at times. Persistent and forceful attempts are necessary to get the child's attention at times. Minimal contact is initiated by the child.

Rating 4: Severely abnormal relationships. The child is constantly aloof or unaware of what the adult is doing. He or she almost never responds or

initiates contact with the adult. Only the most persistent attempts to get the child's attention have any effect.

Category II Imitation:

Rating 1: Appropriate imitation. The child can imitate sounds, words, and movements which are appropriate for his or her skill level.

Rating 2: Mildly abnormal imitation. The child imitates simple behaviors such as clapping or single verbal sounds most of the time: occasionally, imitates only after prodding or a delay.

Rating 3: Moderately abnormal imitation. The child imitates only part of the time and requires a great deal of persistence and help from the adult; frequently imitates only after a delay.

Rating 4: severely abnormal imitation. The child rarely or never imitates sounds, words, or movements even with prodding and assistance.

Category III Emotional response:

Rating 1: Age-appropriate and situation-appropriate responses. The child shows the appropriate type and degree of emotional response as indicated by a change in facial expression, posture, and manner.

Rating 2: Mildly abnormal emotional responses. The child occasionally displays a somewhat inappropriate type or degree of emotional response. Reactions are sometimes unrelated to the objects or event surrounding them.

Rating 3: Moderately abnormal emotional responses. The child shows definite signs of Reactions may be quite inhibited or excessive and unrelated to the situation; may grimace, laugh, or become rigid even though no apparent emotion producing objects or events are present.

Rating 4: Severely abnormal emotional responses. Responses are seldom appropriate to the situation; once the child gets in a certain mood, it is very difficult to change the mood. Conversely, the child may show wildly different emotions when nothing has changed.

Category IV Body Use

Rating 1: Age-appropriate body use. The child moves with the same ease, agility, and coordination of a normal child of the same age.

Rating 2: Mildly abnormal body use. Some minor peculiarities may be present, such as clumsiness, repetitive movements, poor coordination or the rare appearance of more unusual movements.

Rating 3: Moderately abnormal body use. Behaviors that are clearly strange or unusual for a child of this age may include strange finger movements, peculiar finger or body posturing, staring, picking at the body, self-directed aggression, rocking, spinning, or toe walking.

Rating 4: Severely abnormal body use. Intense of frequent movements of the type listed above are signs of severely abnormal body use. The behaviors may persist despite attempts to discourage them or involve the child in other activities.

Category V Object Use:

Rating 1: Appropriate use of, and interest in, toys and other objects. The child shows normal interest in toys and other objects appropriate for his or her skill level and uses these toys in appropriate ways.

Rating 2: Mildly inappropriate use of, and interest in, toys and other objects. The

child may show atypical interest in a toy or play with it in an inappropriate, childish way (e.g. banging or sucking on the toy).

Rating 3: Moderately inappropriate use of, and interest in, toys and other objects. The child may show little interest in toys or other objects, or he may be preoccupied with using an object or toy in some strange way. He or she may focus on some insignificant part of the toy, become fascinated with light reflection off the object, repetitively move some part of the

object, or play with the object exclusively.

Rating 4: Severely inappropriate use of, and interest in, toys and other objects. The child may engage in the same behaviors as above with greater frequency and intensity. The child is difficult to distract when engaged in these inappropriate activities.

Category VI Adaptation to change:

Rating 1: Age-appropriate responses to change. While the child may notice or comment on change in routines, he or she accepts these changes without undue distress.

Rating 2: Mildly abnormal age-appropriate responses to change. When an adult tries to change tasks, the child may continue the same activity or use the same materials.

Rating 3: Moderately abnormal age-appropriate responses to change. The child actively resists changes in routine, tries to continue the old activity, and is difficult to distract. He or she may become angry and unhappy when an established routine is altered.

Rating 4: Severely abnormal age-appropriate responses to change. The child shows

sever reactions to change. If a change is forced, he or she may become extremely angry or uncooperative and respond with tantrums.

Category VII Visual response:

Rating 1: The child's visual behavior is normal and appropriate for that age. Vision is used together with the other senses as a way to explore a new object.

Rating 2: Mildly abnormal visual response. The child must be occasionally reminded to look at objects. The child may be more interested in looking at mirrors or lighting than peers, may occasionally stare off into space, or may avoid looking people in the eye.

Rating 3: Moderately abnormal visual response. The child must be reminded frequently to look at what he or she is doing. He or she may stare off into space, avoid looking people in the eye, look at objects from an unusual angle, or hold objects very close to the eyes.

Rating 4: Severely abnormal visual response. The child consistently avoids looking at people or certain objects and may show extreme forms of other visual peculiarities described above.

Category VIII Listening Response:

Rating 1: Age-appropriate listening responses. The child's listening behavior is normal and appropriate for age. Listening is used together with the other senses.

Rating 2: Mildly abnormal listening responses. There may be some lack of response, or mild overreaction to certain sounds. Responses to sounds may be delayed, and sounds may need repetition to catch the child's attention. The child may be distracted

by extraneous sounds.

Rating 3: Moderately abnormal listening responses. The child's responses to sounds vary; often ignores a sound the first few times it is made; may be startled or cover ears when hearing some everyday sounds

Rating 4: Severely abnormal listening responses. The child overreacts and/or under reacts to sounds to an extremely marked degree, regardless of the type of sound.

Category IX Taste, Smell, and Touch Response and Use:

Rating 1: Normal use of, and response to taste, smell, and touch. The child explores new objects in an age appropriate manner, generally by feeling or looking. Taste or smell may be used when appropriate. When reacting to minor everyday pain, the child expresses discomfort but does not overreact.

Rating 2: Mildly abnormal use of, and responses to, taste, smell, and touch. The child may persist in putting objects in his or her mouth; may smell or taste inedible objects; may ignore or overreact to mild pain that a normal child would express only discomfort.

Rating 3: Moderately abnormal use of, and responses to, taste, smell, and touch. The child may be moderately preoccupied with touching, smelling, or tasting objects or people. The child may react too much or too little.

Rating 4: Severely abnormal use of, and responses to, taste, smell, and touch. The child is preoccupied with smelling, tasting, and feeling objects more for the sensation than for normal exploration or use of the objects. The child may completely ignore pain or react very strongly to slight discomforts.

Category X Fear or Nervousness:

Rating 1: Normal fear or nervousness. The child's behavior is appropriate both to the situation and to his or her age.

Rating 2: Mildly abnormal fear or nervousness. The child occasionally shows too much or too little fear or nervousness compared to the reactions of a normal child of the same age and similar situation.

Rating 3: Moderately abnormal fear or nervousness. The child shows either quite a bit more or quite a bit less fear than is typical for even a child younger or older in a similar situation.

Rating 4: Severely abnormal fear or nervousness. Fears persist even after repeated experience with harmless events or objects. It is extremely difficult to calm or comfort the child. The child may, conversely, fail to show appropriate regard for the hazards other children of the same age avoid.

Category XI Verbal Communication:

Rating 1: Normal verbal communication, age and situation appropriate.

Rating 2: Mildly abnormal verbal communication. Speech shows overall retardation. Most speech is meaningful; however, some echolalia or pronoun reversal may occur. Some peculiar words or jargon may be used occasionally.

Rating 3: Moderately abnormal verbal communication. Speech may be absent. When present, verbal communication may be a mixture of some meaningful communication and some peculiar speech such as jargon, echolalia, or pronoun reversal. Peculiarities in meaningful speech may include excessive questioning or preoccupation with

particular topics.

Rating 4: Severely abnormal verbal communication. Meaningful speech is not used. The child may make infantile squeals, weird or animal-like sounds, complex noises approximating speech, or may show persistent, bizarre use of some recognizable words or phrases

Category XII Nonverbal Communication:

Rating 1: Normal use of nonverbal communication, age and situation appropriate.

Rating 2: Mildly abnormal use of nonverbal communication. Immature use of nonverbal communication; may only point vaguely, or reach for what he or she wants, in same situations where the same-age child may point or gesture more specifically to indicate what he or she wants.

Rating 3: Moderately abnormal use of nonverbal communication. The child is generally unable to express needs or desires nonverbally, and cannot understand the nonverbal communication of others.

Rating 4: Severely abnormal use of nonverbal communication. The child only uses bizarre or peculiar gestures which have no apparent meaning, and shows no awareness of the meanings associated with the gestures or facial expressions of others.

Category XII Activity Level:

Rating 1: Normal activity level for age and circumstances. The child is neither more active nor less active than a normal child of the same age in a similar situation.

Rating 2: Mildly abnormal activity level. The child may either be mildly restless or

somewhat "lazy" and slow moving at times. The child's activity level interferes only slightly with his or her performance.

Rating 3: Moderately abnormal activity level. The child may be quite active and difficult to restrain. He or she may have boundless energy or may not go to sleep readily at night. Conversely, the child may be quite lethargic, and need a great deal of prodding to get him or her to move about.

Rating 4: Severely abnormal activity level. The child exhibits extremes of activity or inactivity and may even shift from one extreme to the other.

Category XIV Level and Consistency of Intellectual Response:

Rating 1: Intelligence is normal and reasonably consistent across various areas. The child is as intelligent as typical children of the same age and does not have any unusual intellectual skills or problems.

Rating 2: Mildly abnormal intellectual functioning. The child is not as smart as typical children of the same age; skills appear fairly evenly across all areas.

Rating 3: Moderately abnormal intellectual functioning. In general, the child is not as smart as typical children of the same age; however, the child may function nearly normally in one or more intellectual areas.

Rating 4: Severely abnormal intellectual functioning. While the child generally is not as smart as the typical child of the same age, he or she may function even better than the normal child of the same age in one or more areas.

Category XV General Impressions:

Rating 1: No Autism: The child shows none of the symptoms characteristic of autism.

Rating 2: Mild Autism. The child shows only a few symptoms or only a mild degree of autism.

Rating 3: Moderate autism. The child shows a number of symptoms or a moderate degree of autism.

Rating 4: Severe autism. the child shows many symptoms or an extreme degree of autism**.**

Supplemental Information 2

Detailed information by each Co-exposure analysis

BKMR: Bayesian Kernel Machine Regression

BKMR is a highly flexible statistical approach that can adeptly model complex relationships. It excels at capturing non-linear and interactive effects among various exposures within a mixture without needing these to be explicitly defined in the model. This method is particularly useful for identifying which specific chemicals in a mixture are most impactful on a health outcome and understanding their combined effects.

WQS: Weighted Quantile Sum Regression

WQS regression is a more constrained method that aims to estimate the overall effect of a chemical mixture. It works by creating a weighted index of the different exposures, where all exposures are assumed to have the same effect direction (either all are adverse or all are protective). The model then provides an estimate of the overall mixture effect and identifies the individual chemicals that contribute most significantly to this combined effect through their assigned weights.

qgcomp: Quantile G-Computation

Quantile G-Computation is a newer method that, like WQS, estimates the overall effect of a mixture. However, a key advantage of qgcomp is that it does not assume that all chemicals have the same directional effect on the outcome. This allows for a more realistic assessment of complex mixtures where some components might be harmful while others could be protective or have no effect. It provides an estimate of the mixture effect for a specific increase in the quantile of all exposures.

\

Supplemental Figure Section

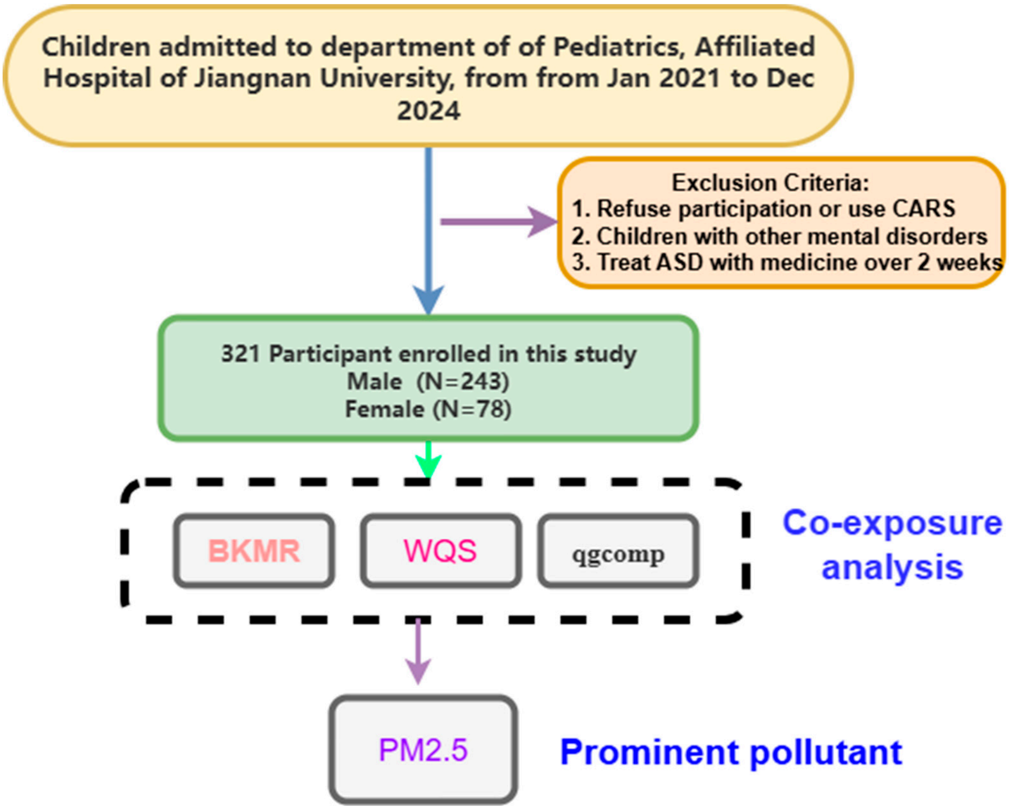

Supplemental Figure 1: The flowchart of observational cohort in this study

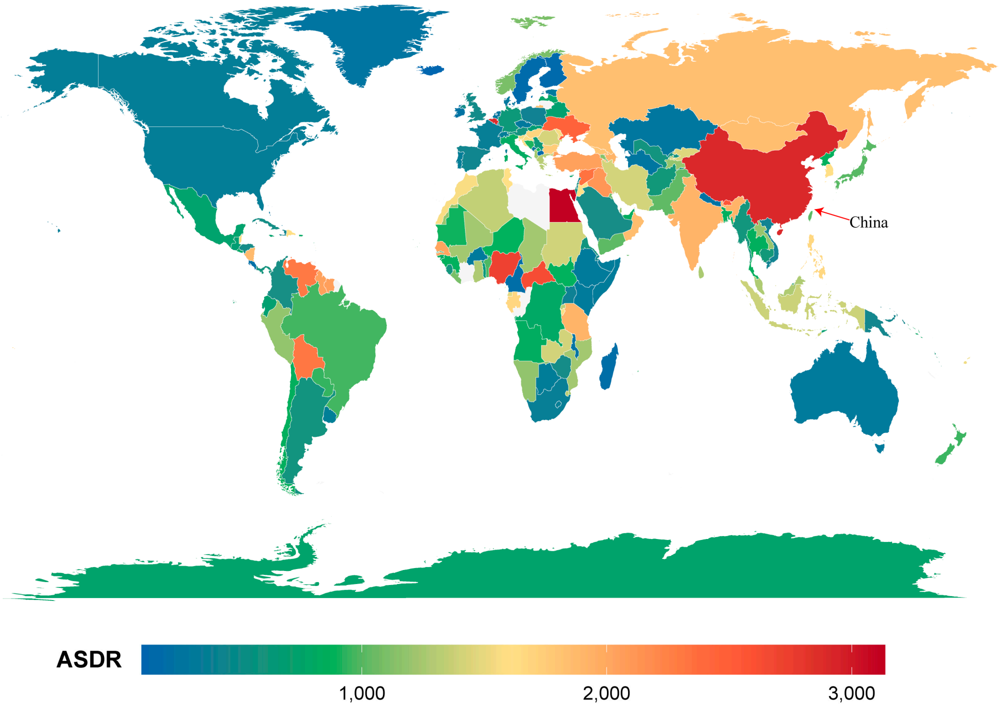

Supplemental Figure 2: The flowchart of observational cohort in this study

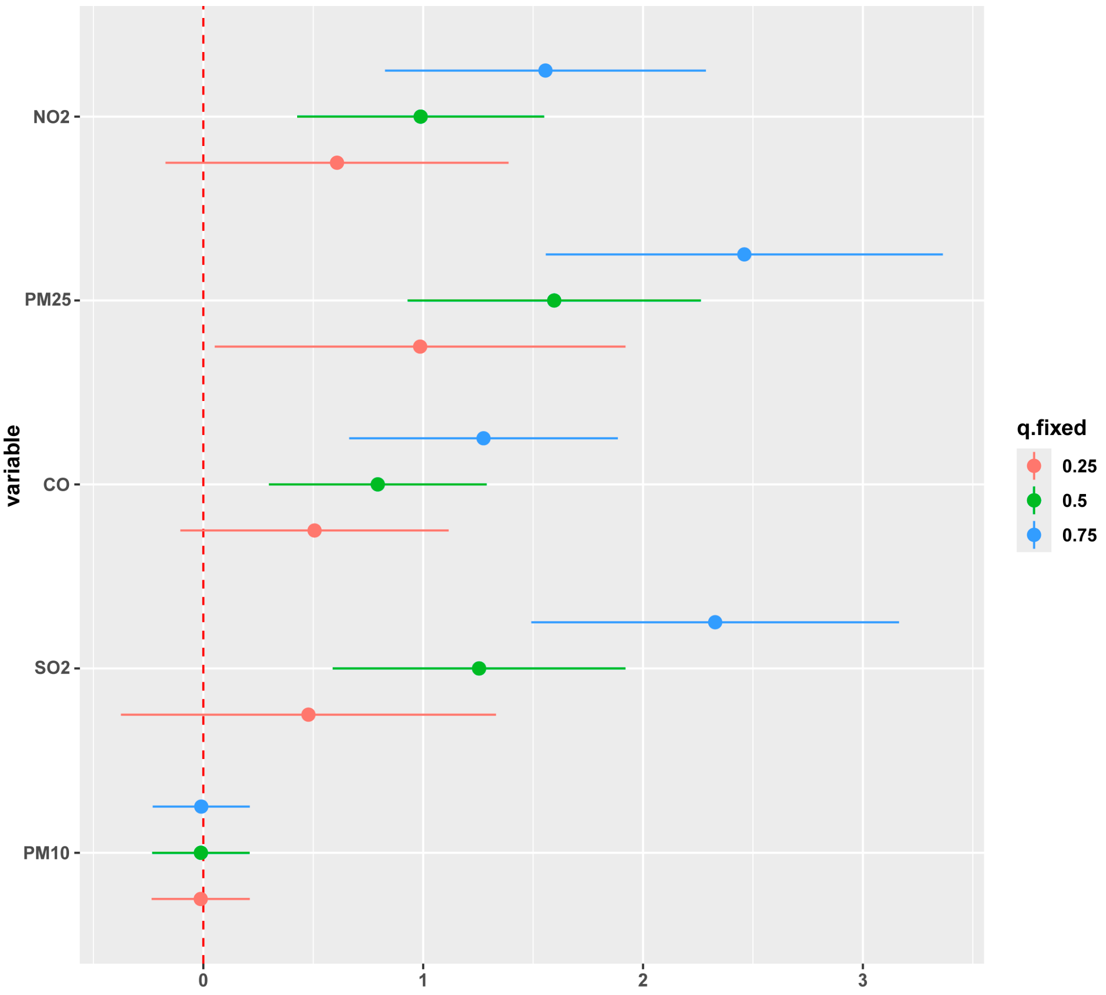

Supplemental Figure 3: The BKMR single-exposure effect plot

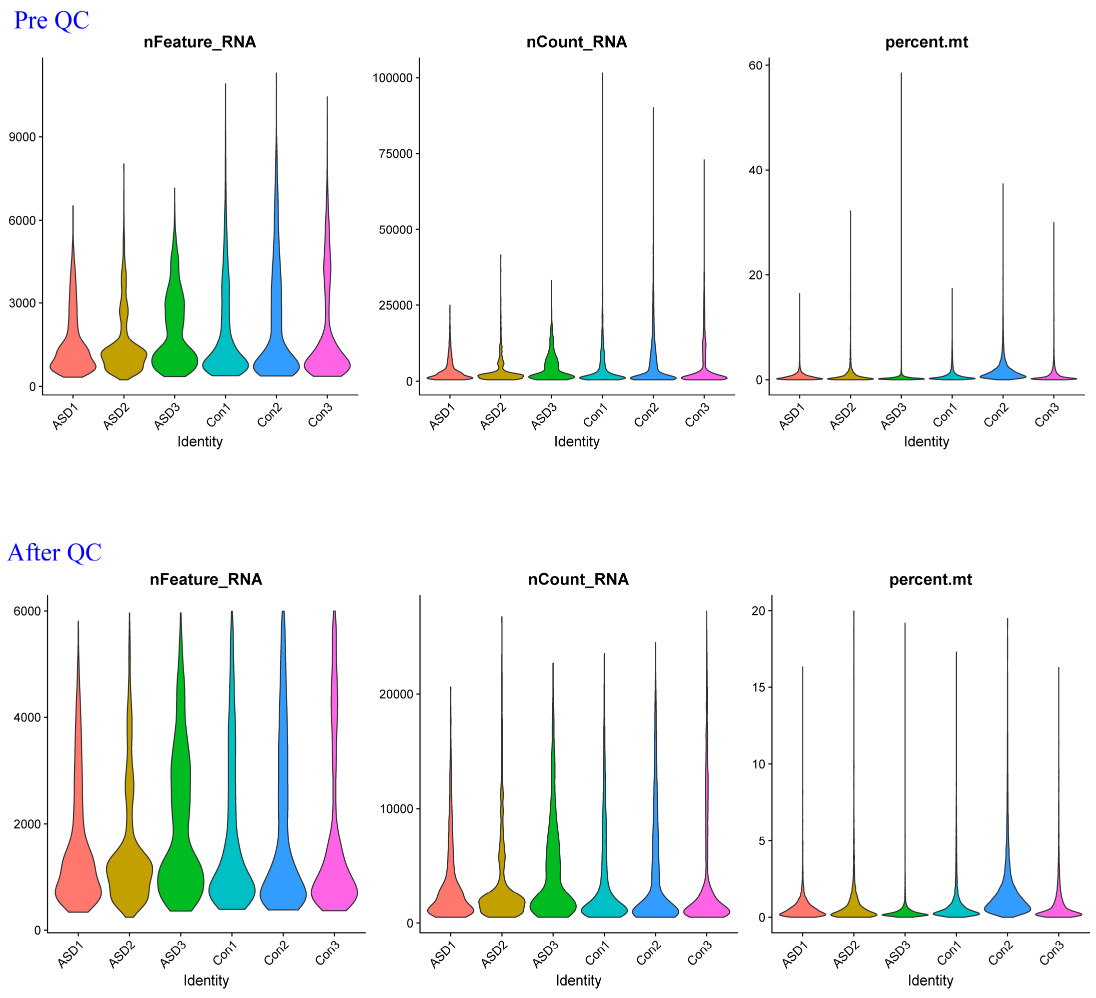

Supplemental Figure 4: Data Quality Control Before and After Filtering in scRNA-seq Analysis

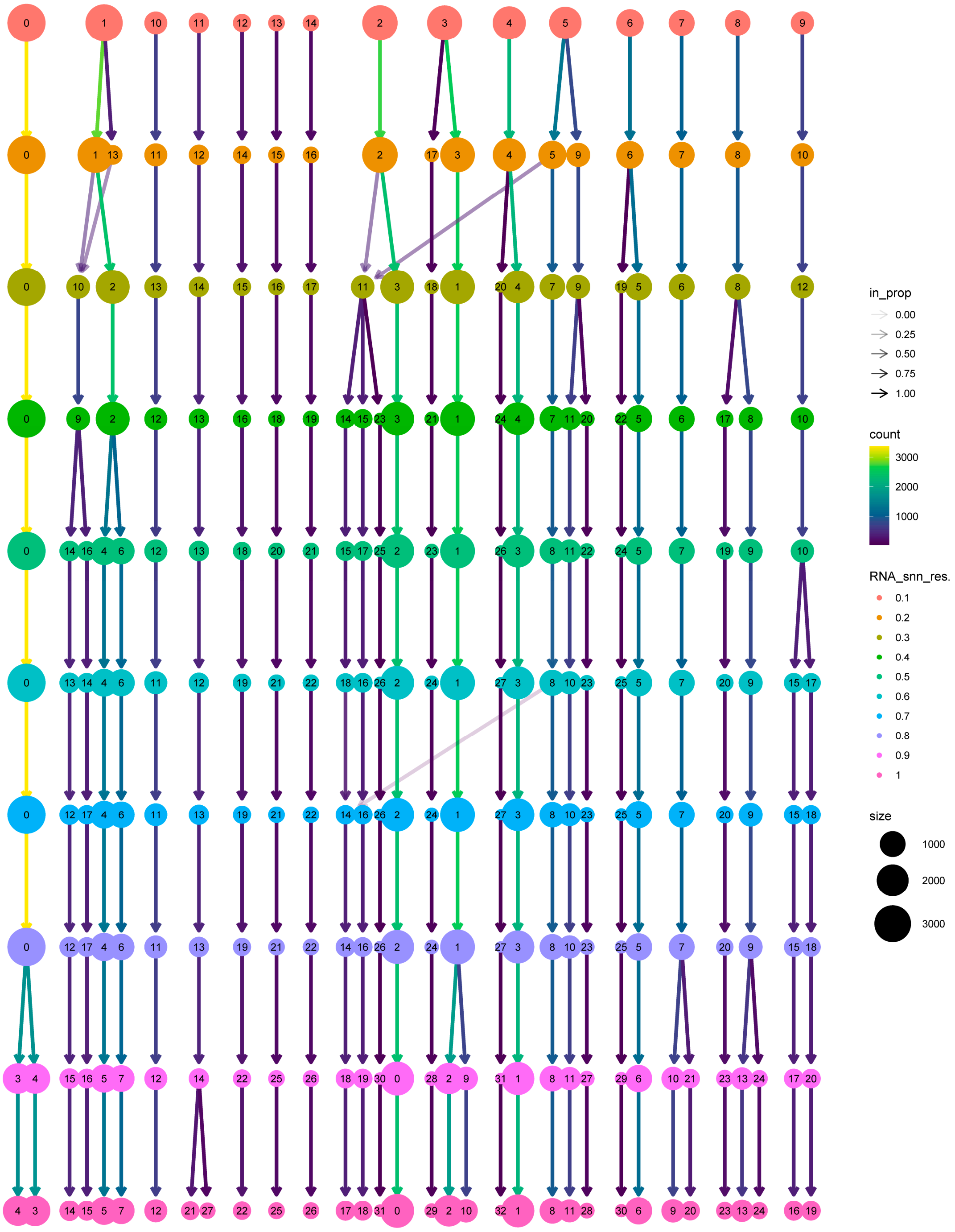

Supplementary Figure 5: Clustering Tree for scRNA-seq Analysis

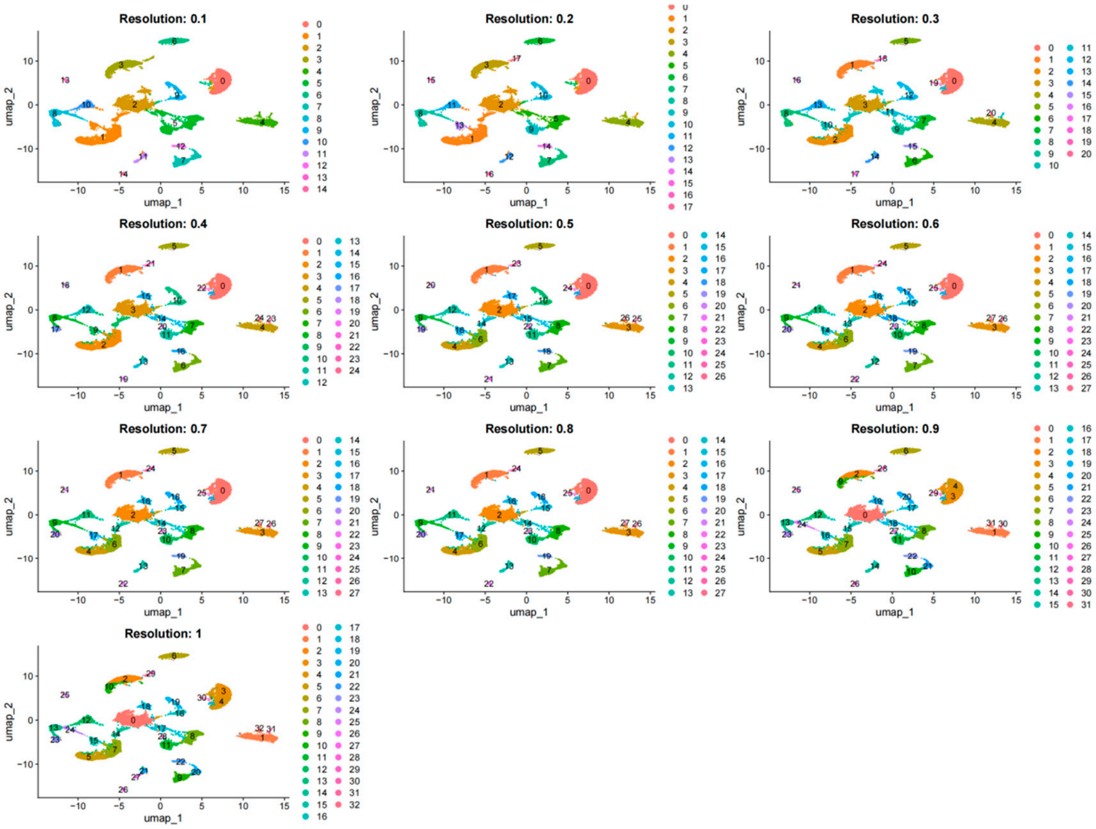

Supplementary Figure 6: UMAP Clustering at Different Resolutions

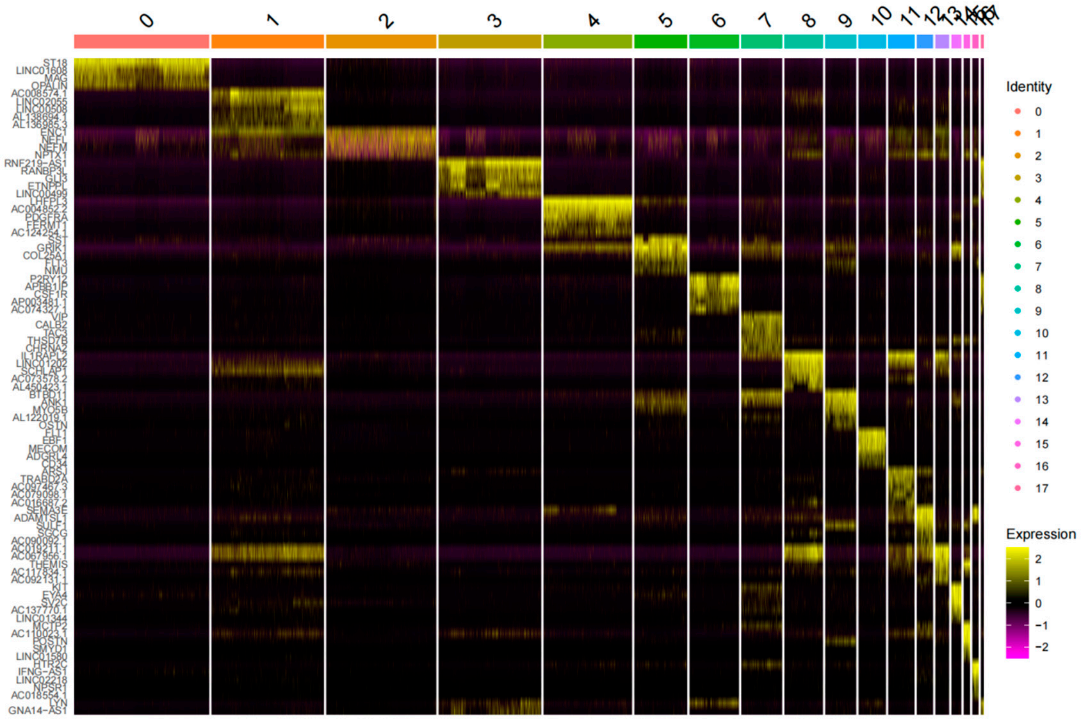

Supplementary Figure 7: Heatmap of genes Markers Across All Cluster

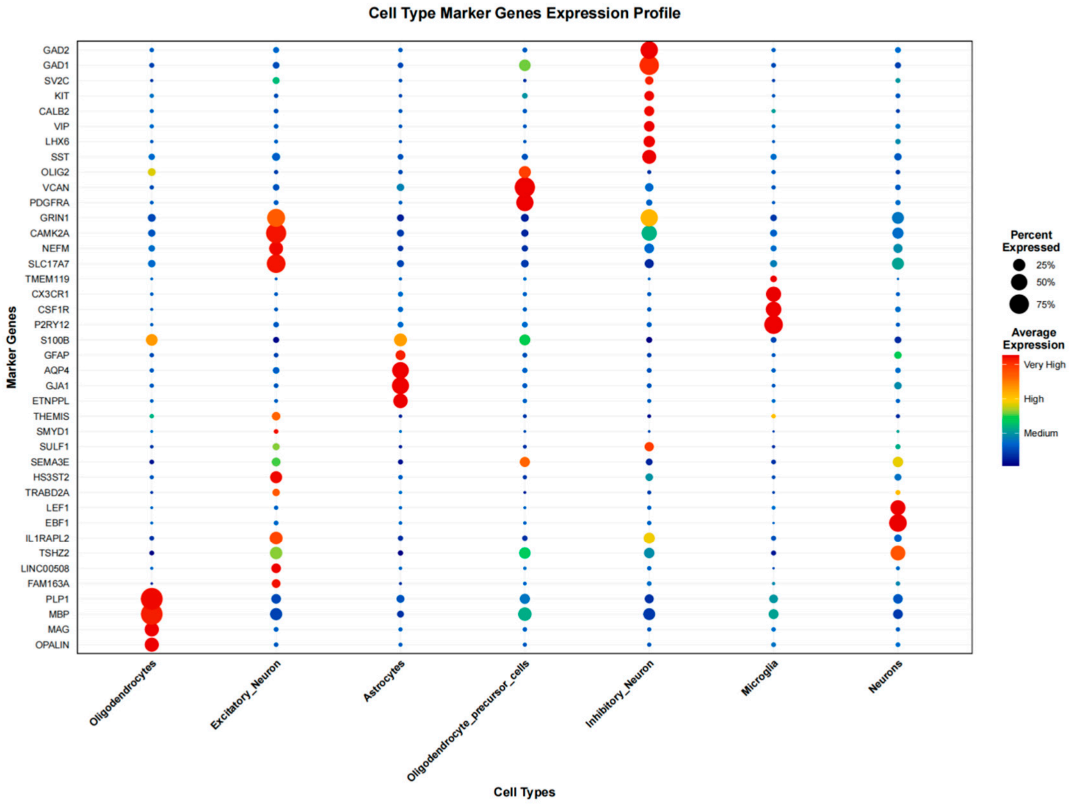

Supplementary Figure 8: Dotplot of Genes Markers Across All Cell Types

Supplementary Methods Section

1. Mendelian Randomization analysis

Mendelian Randomization (MR) analysis is a powerful technique that leverages genome-wide association studies (GWAS) data by using genetic variants as instrumental variables (IVs) to explore potential relationships between risk factors and health outcomes[1]. MR analysis has been widely employed to assess the effects of environmental exposures, such as air pollution, on disease risk[2]. In this study, we aimed to identify risk factors highly associated with ASD symptoms and establish the association between air pollution exposure-induced gene expression alterations and ASD severity[3, 4]. In brief, we collected GWAS data of common air pollutants from the IEU OpenGWAS project and ASD severity risk data from the Psychiatric Genomics Consortium (Supplementary Table 3). The objective of this study was to explore the potential associations between air pollution exposure and ASD severity risk. To achieve this, we selected instrumental variables (IVs) using a significance threshold of $P < 1.0 \times 10^{-5}$. This threshold was chosen based on previous research indicating its effectiveness in identifying genetic predictors that explain significant variance in the outcome. We employed Inverse Variance Weighted Mendelian Randomization (IVW) as our primary analytical method to ensure robustness and address potential pleiotropy in the instrumental variables.

2. RNA sequencing analysis

RNA sequencing analysis was conducted following the procedures described in our previous report. In brief, The data was obtained from the GEO database of GSE18123.

Differentially expressed genes (DEGs) were identified based on the criteria of fold change ≥ 1.5 and P-value less than 0.05. To visualize the expression patterns, heatmaps and volcano plots were conducted based on our previous study. For functional interpretation, Gene Ontology (GO) enrichment analysis and Kyoto Encyclopedia of Genes and Genomes (KEGG) pathway analysis were performed on the DEGs. These analyses provided insights into the biological processes, molecular functions, cellular components, and signaling pathways significantly associated with the observed gene expression changes.

3. Acquisition of potential targets and therapeutic agent analysis

To identify potential human gene targets associated with the impact of $PM_{2.5}$ exposure-induced neurotoxicity on seizure severity, we employed a network toxicology methodology based on our previous research. The primary steps of this approach included: (1) Identification of Gene Targets Linked to $PM_{2.5}$ Exposure via CTD: We began by retrieving potential human gene targets related to $PM_{2.5}$ exposure from the Comparative Toxicogenomics Database (CTD, https://ctdbase.org). CTD is a comprehensive cross-species database that compiles information on chemical exposures and their biological effects. For this analysis, we utilized the keywords "$PM_{2.5}$". (2) Extraction of ASD-Associated Gene Targets from Gene Cards: Concurrently, we extracted gene targets associated with seizures from the Gene Cards Database (https://genecards.org). Gene Cards provides detailed information on the genomic, proteomic, transcriptomic, genetic, and functional aspects of all documented human genes. The search was conducted using the keyword "autism". (3) Intersection

Analysis Using Venn Diagram: A Venn Diagram was utilized to identify overlapping genes between the datasets obtained from CTD and Gene Cards, highlighting the intersecting gene targets relevant to both $PM_{2.5}$ exposure and ASD. (4) Pathway Enrichment Analysis via Metascape: The intersecting genes were further analyzed for their involvement in biological pathways using Gene Ontology (GO) and Kyoto Encyclopedia of Genes and Genomes (KEGG) pathways through the Metascape Database (https://metascape.org). (5) Construction of Protein-Protein Interaction (PPI) Networks: PPI networks for the overlapping genes were constructed using the STRING database (https://cn.string-db.org). Subsequently, the CytoHubba plugin within Cytoscape (https://cytoscape.org/) was employed to identify the top 20 hub genes.

4. Three-box social interaction test

The three-box social interaction test is a behavioral assay used to evaluate sociability and social novelty preference in rodents, particularly in the context of CNS disorders like autism[5]. In this investigation, we employed the three-box social interaction test to explore the potential effects of $PM_{2.5}$ exposure on social behaviors and social behavior deficits in ASD mice, following the methodology described in a previous study[5]. Briefly, the experimental setup consists of a rectangular box divided into three chambers with removable partitions, allowing the test animal to move freely between the chambers. During the habituation phase, mice were allowed to explore the three-chamber apparatus for 10 minutes. In the sociability test, one of the side chambers contained a wire cage with a stranger mouse (Stranger 1), while the other

side chamber contained an empty cage. The test mice were given 10 minutes to explore the apparatus and interact with either the empty cage or Stranger 1. In the social novelty preference test, a second stranger mouse (Stranger 2) was introduced into the previously empty cage, and the test mice were given another 10 minutes to explore the apparatus and interact with the stranger mice. Sociability and social novelty preference were assessed by manually recording the time spent by the test mouse actively interacting with Stranger 1 or Stranger 2 during the sociability and social novelty phases, respectively.

5. Novel object recognition (NOR) test

To investigate and confirm the potential effects of $PM_{2.5}$ exposure on short-term recognition memory in ASD mice, we utilized the NOR test, as detailed in our previous studies [3, 4, 6, 7]. Specifically, we meticulously recorded the duration each mouse spent exploring the familiar object (F) compared to the novel object (N). The discrimination index (DI) was then calculated using the formula: $(N-F)/(N+F) \times 100\%$. This metric enabled us to assess the mice's ability to differentiate between familiar and novel objects, thereby evaluating their recognition memory performance.

6. Marble burying test

The marble burying test is commonly used to assess anxiety-like behavior in rodents [8]. In this study, we conducted the marble burying test to explore the effects of $PM_{2.5}$ exposure on anxiety-like behaviors in ASD mice, in alignment with the methodology of our previous study [4]. In brief, the mice were placed in a standard laboratory cage containing a 5 cm-thick layer of bedding material. Before the

experiment, twelve colored glass marbles were laid out on the surface of the bedding in a grid pattern [9]. During the 30-minute test, the mice were allowed to move freely within the cage. Only those marbles that were covered by at least two-thirds with sawdust were considered "buried." A higher number of buried marbles may indicate increased anxiety-like behaviors or obsessive-compulsive tendencies [9, 10].

21. Quantitative real-time reverse transcription PCR (qRT-PCR)

To examine the effects of $PM_{2.5}$ exposure on gene expression, particularly the change of CTNNB1, PTEN, CCR2, AKT1, and mTOR. We conducted qRT-PCR following the protocols established in our previous studies. Initially, total RNA was isolated from leukocytes in human blood samples using the MolPure® Blood RNA Kit (Catalog No. 19241ES50, YEASEN, Shanghai, China) and from hippocampal tissues utilizing TRIzol reagent (Takara, Shiga, Japan) as per the manufacturer's instructions. The purified RNA was then reverse-transcribed into complementary DNA (cDNA). Relative mRNA expression levels were determined using the 2^-(ΔΔCt) method, with normalization performed against the housekeeping gene glyceraldehyde 3-phosphate dehydrogenase (GAPDH). Detailed primer sequences employed for amplification are provided in Supplementary Table 4.

Reference

1. Mei H, Wu D, Yong Z, Cao Y, Chang Y, Liang J*, et al.* PM(2.5) exposure exacerbates seizure symptoms and cognitive dysfunction by disrupting iron metabolism and the Nrf2-mediated ferroptosis pathway. **Sci Total Environ 2024**, 910**:** 168578.
2. Cao Y, Zhao W, Zhong Y, Jiang X, Mei H, Chang Y*, et al.* Effects of chronic low-level lead (Pb) exposure on cognitive function and hippocampal neuronal ferroptosis: An integrative approach using bioinformatics analysis, machine learning, and experimental validation. **Sci Total Environ 2024**, 917**:** 170317.

3. Chang Y, Jiang X, Dou J, Xie R, Zhao W, Cao Y, *et al.* Investigating the potential risk of cadmium exposure on seizure severity and anxiety-like behaviors through the ferroptosis pathway in epileptic mice: An integrated multi-omics approach. **J Hazard Mater 2024**, 480**:** 135814.
4. Hu J, Xu J, Li M, Jiang Z, Mao J, Feng L, *et al.* Identification and validation of an explainable prediction model of acute kidney injury with prognostic implications in critically ill children: a prospective multicenter cohort study. **EClinicalMedicine 2024**, 68**:** 102409.
5. Sanderson E, Glymour MM, Holmes MV, Kang H, Morrison J, Munafò MR, *et al.* Mendelian randomization. **Nat Rev Methods Primers 2022**, 2.
6. Lin L, Zhang R, Huang H, Zhu Y, Li Y, Dong X, *et al.* Mendelian Randomization With Refined Instrumental Variables From Genetic Score Improves Accuracy and Reduces Bias. **Front Genet 2021**, 12**:** 618829.
7. Zhu M, Li X, Lin W, Zeng D, Yang P, Ni W, *et al.* Microplastic Particles Detected in Fetal Cord Blood, Placenta, and Meconium: A Pilot Study of Nine Mother-Infant Pairs in South China. **Toxics 2024**, 12(12).
8. Prata JC, da Costa JP, Fernandes AJS, da Costa FM, Duarte AC, Rocha-Santos T. Selection of microplastics by Nile Red staining increases environmental sample throughput by micro-Raman spectroscopy. **Sci Total Environ 2021**, 783**:** 146979.
9. Zheng X, Feng Q, Chen J, Yan J, Li X, Guo L. Quantification analysis of microplastics released from disposable polystyrene tableware with fluorescent polymer staining. **Sci Total Environ 2023**, 864**:** 161155.
10. Wang Y, Quan F, Cao Q, Lin Y, Yue C, Bi R, *et al.* Quercetin alleviates acute kidney injury by inhibiting ferroptosis. **J Adv Res 2021**, 28**:** 231–243.
11. Singh S, Botvinnik A, Shahar O, Wolf G, Yakobi C, Saban M, *et al.* Effect of psilocybin on marble burying in ICR mice: role of 5-HT1A receptors and implications for the treatment of obsessive-compulsive disorder. **Transl Psychiatry 2023**, 13(1)**:** 164.
12. Horii-Hayashi N, Masuda K, Kato T, Kobayashi K, Inutsuka A, Nambu MF, *et al.* Entrance-sealing behavior in the home cage: a defensive response to potential threats linked to the serotonergic system and manifestation of repetitive/stereotypic behavior in mice. **Front Behav Neurosci 2023**, 17**:** 1289520.
13. Samra AI, Kamel AS, Abdallah DM, El Fattah MAA, Ahmed KA, El-Abhar HS. Preclinical Evidence for the Role of the Yin/Yang Angiotensin System Components in Autism Spectrum Disorder: A Therapeutic Target of Astaxanthin. **Biomedicines 2023**, 11(12).
14. Dang R, Wang M, Li X, Wang H, Liu L, Wu Q, *et al.* Edaravone ameliorates depressive and anxiety-like behaviors via Sirt1/Nrf2/HO-1/Gpx4 pathway. **J Neuroinflammation 2022**, 19(1)**:** 41.
15. Lee JH, Yun JY, Gregory A, Hogarth P, Hayflick SJ. Brain MRI Pattern Recognition in Neurodegeneration With Brain Iron Accumulation. **Front Neurol 2020**, 11**:** 1024.
16. Xie R, Zhao W, Lowe S, Bentley R, Hu G, Mei H, *et al.* Quercetin alleviates kainic acid-induced seizure by inhibiting the Nrf2-mediated ferroptosis pathway. **Free Radic Biol Med 2022**, 191**:** 212–226.
17. Xie R, Li T, Qiao X, Mei H, Hu G, Li L, *et al.* The Protective Role of E-64d in Hippocampal Excitotoxic Neuronal Injury Induced by Glutamate in HT22 Hippocampal Neuronal Cells. **Neural Plast 2021**, 2021**:** 7174287.

18. Xie Q, Hu B. Effects of gut microbiota on prostatic cancer: a two-sample Mendelian randomization study. **Front Microbiol 2023**, 14**:** 1250369.

Supplemental Table Section

Supplemental Table 1

The World Health Organization's (WHO) new World Standard Population

Age Group	WHO World Standard (%)	Recalculation to add to 1,000,000
0-4	8.86	88569.0008497026
5-9	8.69	86869.5956415255
10-14	8.6	85969.910531314
15-19	8.47	84670.3653721198
20-24	8.22	82171.2400659769
25-29	7.93	79272.2547108512
30-34	7.61	76073.3743189884
35-39	7.15	71474.9837556855
40-44	6.59	65876.9430699255
45-49	6.04	60378.8673964113
50-54	5.37	53681.2115759484
55-59	4.55	45484.0805717999
60-64	3.72	37186.9845554056
65-69	2.96	29589.6436247313
70-74	2.21	22092.2677063028
75-79	1.52	15194.6818613485
80-84	0.91	9096.81611435998
85-89	0.44	4398.46053881142
90-94	0.15	1499.47518368571
95-99	0.04	399.860048982856
100+	0.005	49.982506122857
Total	100.035	1000000

Supplemental Table 2

The basic characteristics of patients diagnosed with ASD involving in this study

**Characteristic**	**Overall** N = 321[1]	**Male** N = 243[1]	**Female** N = 78[1]	**P-value**[2]
**Age**	3.48 (0.88)	3.48 (0.88)	3.48 (0.89)	>0.9
**BMI**	15.46 (1.45)	15.53 (1.42)	15.25 (1.52)	0.14
**Race**				0.13
Han people	300 (93%)	230 (95%)	70 (90%)	
Others	21 (6.5%)	13 (5.3%)	8 (10%)	
**CARS Score**	34.71 (1.75)	34.72 (1.85)	34.67 (1.38)	0.7
**Education level**				0.5
Preschool	212 (66%)	163 (67%)	49 (63%)	
Kindergarten	109 (34%)	80 (33%)	29 (37%)	

[1]Mean (SD); n (%)

[2]Wilcoxon rank sum test; Pearson's Chi-squared test

Supplemental Table 3

Detailed information of GWAS data used in the study

GWASID	Years	Trait	Sample Size	Population	Database/PMID*
ASD2019	2019	Autism spectrum disorder	46,351	European	Psychiatric Genomics Consortium PMID:30804558
ukb-b-10817	2010	$PM_{2.5}$	423,796	European	IEU OPEN GWAS NA
ukb-b-12963	2010	$PM_{2.5}$-PM_{10}	423,796	European	IEU OPEN GWAS NA
ukb-b-18469	2010	PM_{10}	423,796	European	IEU OPEN GWAS NA
ukb-b-12417	2010	NO_X	423,796	European	IEU OPEN GWAS NA

*IEU OPEN GWAS (https://gwas.mrcieu.ac.uk/)

*Psychiatric Genomics Consortium (https://pgc.unc.edu/)

Supplemental Table 4

Detailed information of primer sequences utilized in this study

Name	Sequence
CTNNB1 forward	5′-ATGGAGCCGGACAGAAAAGC-3′
CTNNB1 reverse	5′-TGGGAGGTGTCAACATCTTCTT-3′
PTEN forward	5′-TGGATTCGACTTAGACTTGACCT-3′
PTEN reverse	5′-GCGGTGTCATAATGTCTCTCAG-3′
CCR2 forward:	5′-ATCCACGGCATACTATCAACATC-3′
CCR2 reverse:	5′-TCGTAGTCATACGGTGTGGTG-3′
AKT1 forward:	5′-ATGAACGACGTAGCCATTGTG-3′
AKT1 reverse:	5′-TTGTAGCCAATAAAGGTGCCAT-3′
mTOR forward	5′-GCTTTGACGCAGGTGCTAAG-3′
mTOR reverse	5′-TGTCCCCATAACCGGAGTAGG -3′
GAPDH forward:	5′- AGGTCGGTGTGAACGGATTTG -3′
GAPDH reverse:	5′- GGGGTCGTTGATGGCAACA -3′
